# Supplementary material for: Public knowledge, practices, and awareness of antibiotics and antibiotic resistance in Myanmar: The first national mobile phone panel survey
Source: PLoS One. 2022 Aug 18;17(8):e0273380. doi: 10.1371/journal.pone.0273380 (PMC9387831; doi:10.1371/journal.pone.0273380)
Supplement: S1 File — (DOCX) [file pone.0273380.s001.docx]

**Annex 1-1. Survey questionnaire (English)**

| 1. AWARENESS AND USAGE OF ANTIBIOTICS | |
| --- | --- |
| A1. Do you know “antibiotics”? | 1. YES 2. NO |
| A2. If yes in A1, have you taken any antibiotics? If yes, when did you take antibiotics last time? | 1.YES 2. NO  _________________ |
| A3. If yes in A2, where did you acquire the last course of antibiotics? | 1. Clinics 2. Medical store or pharmacy 3. Stall or hawker 4. Friend or family member 5. I had them saved up from a previous time 6. Somewhere/someone else   99. Do not remember |
| A4. If yes in A2, did you show a prescription when you purchased the antibiotics? | 1. YES 2. NO |
| A5. If yes in A4, from whom did you obtain the prescription? | 1. Doctor 2. Pharmacist 3. Others (specify____________)   99. Do not remember |
| A6. When did you stop taking antibiotics once you started a course of treatment? | 1. When you feel better 2. When you’ve taken all of the antibiotics as directed   99. Don’t know |
| B. KNOWLEDGE ABOUT ANTIBIOTICS | |
| B1. Antibiotics kill viruses | 1. TRUE 2. FALSE 99. DON’T KNOW |
| B2. Antibiotics are effective against colds and flu | 1. TRUE 2. FALSE 99. DON’T KNOW |
| B3. Unnecessary use of antibiotics makes them become ineffective | 1. TRUE 2. FALSE 99. DON’T KNOW |
| B4. Taking antibiotics often has side effects such as diarrhea | 1. TRUE 2. FALSE 99. DON’T KNOW |
| B5. Antibiotics are not equal to painkillers or antipyretics | 1. TRUE 2. FALSE 99. DON’T KNOW |
| C. KNOWLEDGE ABOUT ANTIBIOTIC RESISTANCE | |
| C1. Have you ever received any information that you should not take antibiotics unnecessarily for illnesses such as cold or flu, or information on antibiotic resistance? | 1. YES 2. NO |
| C2. If yes in C1, how did you receive the information? | 1. Doctor or nurse 2. Pharmacist 3. Family member or friend 4. Media (newspaper, TV, radio, social media such as Facebook, posters etc.) 5. Specific campaign 6. Others   99. Do not remember |
| C3. If yes in C1, did the information you received change your views on using antibiotics? | 1. YES 2. NO |
| D. AWARENESS OF ANTIBIOTIC USE IN FARM ANIMALS | |
| D1. Did you know that sick farm animals (poultry, pig, and aquaculture) are treated with antibiotics? | 1. YES 2. NO |
| D2. Did you know that using antibiotics in animals can develop antibiotic resistance among them? | 1. YES 2. NO |
| D3. Did you know that using antibiotics to stimulate growth in livestock is banned by Myanmar government? | 1. YES 2. NO |

**Annex 1-2. Survey questionnaire (Burmese)**

| က။ ပဋိဇီဝဆေးဆိုင်ရာအသိပညာနှင့် အသုံးပြုခြင်း | |
| --- | --- |
| က၁။ ပဋိဇီဝဆေး (သို့) လူများ နေမကောင်းဖြစ်လျှင် သောက်သည့် ပိုးသတ်ဆေး ကို ကြားဖူးပါသလား။ | ၁။ ကြားဖူးပါသည်  ၂။ မကြားဖူးပါ |
| က၂။ အကယ်၍ (က၁)တွင် ကြားဖူးပါသည်ဟု ဖြေထားပါက ပဋိဇီဝဆေးကို သောက်ခဲ့ဖူးပါသလား။  သောက်ဖူးပါက ဘယ်အချိန်က ပဋိဇီဝဆေးကို နောက်ဆုံးသောက်ခဲ့ပါသနည်း။ | ၁။ သောက်ဖူးပါသည်  ၂။ မသောက်ဖူးပါ  ___________________ |
| က၃။ အကယ်၍ (က၂)တွင် သောက်ဖူးပါသည်ဟု ဖြေထားပါက နောက်ဆုံးသောက်ခဲ့ဖူးသော ပဋိဇီဝဆေးကို မည်သည့်နေရာမှ ရရှိခဲ့ပါသနည်း။ | ၁။ ဆေးရုံ၊ ဆေးခန်းများ  ၂။ ဆေးဆိုင်၊ ဆေးပေးခန်း  ၃။ ကွမ်းယာဆိုင်၊ စပ်ဆေး  ၄။ သူငယ်ချင်း၊ မိသားစု  ၅။ အရင်တုန်းကသောက်ခဲ့ပြီး ကျန်သောဆေး  ၆။ အခြားတစ်နေရာ၊ တစ်စုံတစ်ယောက်ဆီ မှတစ်ဆင့်  ၉၉။ မမှတ်မိပါ။ |
| က၄။ အကယ်၍ (က၂)တွင် သောက်ဖူးပါသည်ဟု ဖြေထားပါက ပဋိဇီဝဆေးကိုဝယ်သောအခါ ဆေးညွှန်းကိုပြပါသလား။ | ၁။ ပြပါသည်  ၂။ မပြပါ |
| က၅။ အကယ်၍ (က၄)တွင် ပြပါသည်ဟု ဖြေ ထား ပါက ဆေးညွှန်းကို မည်သူဆီကရခဲ့ပါသနည်း။ | ၁။ ဆရာဝန်  ၂။ ဆေးဝါးပညာရှင်  ၃။ အခြား (ဖော်ပြပါ......)  ၉၉။ မမှတ်မိပါ |
| က၆။ ပဋိဇီဝဆေးများသောက်လျှင် မည်သည့်အချိန်တွင် ရပ်သင့်သည်ဟု ထင်သနည်း။ | ၁။ သက်သာသွားသည့်အချိန်  ၂။ ညွှန်ကြားထားသည့်အတိုင်း ဆေးအကုန်သောက်ပြီးလျှင်  ၉၉။ မသိပါ |
| ခ။ ပဋိဇီဝဆေးနှင့်ပတ်သက်သည့် ဗဟုသုတ | |
| ခ၁။ “ပဋိဇီဝဆေးများသည် ဗိုင်းရပ်စ်ပိုးများကို သေစေနိုင်သည်” | ၁။ မှန်သည်  ၂။ မှားသည်  ၉၉။ မသိပါ |
| ခ၂။ “ပဋိဇီဝဆေးများသည် အအေးမိဖျားနာခြင်း၊ တုပ်ကွေးမိခြင်းများအတွက် ထိရောက်မှုရှိသည်” | ၁။ မှန်သည်  ၂။ မှားသည်  ၉၉။ မသိပါ |
| ခ၃။ “မလိုအပ်ပဲ ပဋိဇီဝဆေးများ သုံးစွဲခြင်းသည် ဆေးအာနိသင်ကိုကျစေသည်” | ၁။ မှန်သည်  ၂။ မှားသည်  ၉၉။ မသိပါ |
| ခ၄။ “ပဋိဇီဝဆေးများသောက်လျှင် ဝမ်းလျှောခြင်း ကဲ့သို့ ဆိုးကျိုးများ မကြာခဏရရှိနိုင်သည်” | ၁။ မှန်သည်  ၂။ မှားသည်  ၉၉။ မသိပါ |
| ခ၅။ “ပဋိဇီဝဆေးများသည် အကိုက်အခဲပျောက်ဆေး၊ အဖျားကျဆေးများနှင့် မတူညီပါ” | ၁။ မှန်သည်  ၂။ မှားသည်  ၉၉။ မသိပါ |
| ဂ။ ပဋိဇီဝဆေးယဉ်ပါးမှုနှင့် ပတ်သက်သည့် ဗဟုသုတ | |
| ဂ၁။ ပဋိဇီဝဆေးကို မလိုအပ်သောအချိန်တွင် (ဥပမာ အအေးမိဖျားနာခြင်း၊ တုပ်ကွေးမိခြင်းများတွင်) မသောက်သုံးသင့်ကြောင်းကို သင်ကြားဖူးပါသလား။  (သို့) ပဋိဇီဝဆေးယဉ်ပါးမှု အကြောင်း သင်ကြားဖူးပါသလား။ | ၁။ ကြားဖူးပါသည်  ၂။ မကြားဖူးပါ |
| ဂ၂။ အကယ်၍ (ဂ၁) တွင် ကြားဖူးပါသည် ဟုဖြေထားပါက ပဋိဇီဝဆေးကို မလိုအပ်သောအချိန်တွင် မသောက်သုံးသင့်ကြောင်းကို သင်မည်ကဲ့သို့သိရှိခဲ့သနည်း။ | ၁။ ဆရာဝန်၊ သူနာပြု  ၂။ ဆေးဝါးပညာရှင်  ၃။ မိသားစုဝင် (သို့) သူငယ်ချင်းများ  ၄။ သတင်းမီဒီယာ (သတင်းစာ၊ တီဗီ၊ ရေဒီယို၊ facebook ကဲ့သို့လူမှုကွန်ယက်၊ နံရံကပ် ပိုစတာများ )  ၅။ ပဋိဇီဝဆေးနှင့်ဆိုင်သော လှုပ်ရှားမှု တစ်ခုခု  ၆။ အခြား  ၉၉။ မမှတ်မိပါ |
| ဂ၃။ အကယ်၍ (ဂ၁) တွင် ကြားဖူးပါသည် ဟုဖြေထားပါက သင်ရရှိခဲ့သော သတင်းအချက်အလက်များသည် သင်၏ ပဋိဇီဝဆေးသုံးစွဲပုံကို ပြောင်းလဲစေနိုင်ခဲ့သလား။ | ၁။ ပြောင်းလဲစေနိုင်ခဲ့ပါသည်  ၂။ မပြောင်းလဲစေနိုင်ခဲ့ပါ |
| ဃ။ စိုက်ပျိုးမွေးမြူရေးတွင် ပဋိဇီဝဆေး အသုံးပြုမှု | |
| ဃ၁။ စားသုံးရန်မွေးမြူထားသော တိရစ္ဆာန်များ (ကြက်၊ ဘဲ၊ ဝက်၊ ရေသတ္တဝါများ) နေမကောင်းဖြစ်ပါက ပဋိဇီဝဆေးများနှင့် ကုသနေကြသည်ကို သိရှိပါသလား။ | ၁။ သိပါသည်  ၂။ မသိပါ |
| ဃ၂။ ၎င်းတိရစ္ဆာန်များကို ပဋိဇီဝဆေးများနှင့် ကုသခြင်းသည် ဆေးယဉ်ပါးမှုဖြစ်စေနိုင်သည်ကို သိရှိပါသလား။ | ၁။ သိပါသည်  ၂။ မသိပါ |
| ဃ၃။ ၎င်းတိရစ္ဆာန်များ ကြီးထွားစေရန် ပဋိဇီဝဆေးများ အသုံးပြုခြင်းကို အစိုးရမှ တားမြစ်ထားသည်ကို သိရှိပါသလား။ | ၁။ သိပါသည်  ၂။ မသိပါ |
