# Supplementary material for: Public knowledge, practices, and awareness of antibiotics and antibiotic resistance in Myanmar: The first national mobile phone panel survey
Source: PLoS One. 2022 Aug 18;17(8):e0273380. doi: 10.1371/journal.pone.0273380 (PMC9387831; doi:10.1371/journal.pone.0273380)
Supplement: Attachment — Submitted filename: CATI-130521-en.pdf [file pone.0273380.s004.pdf]

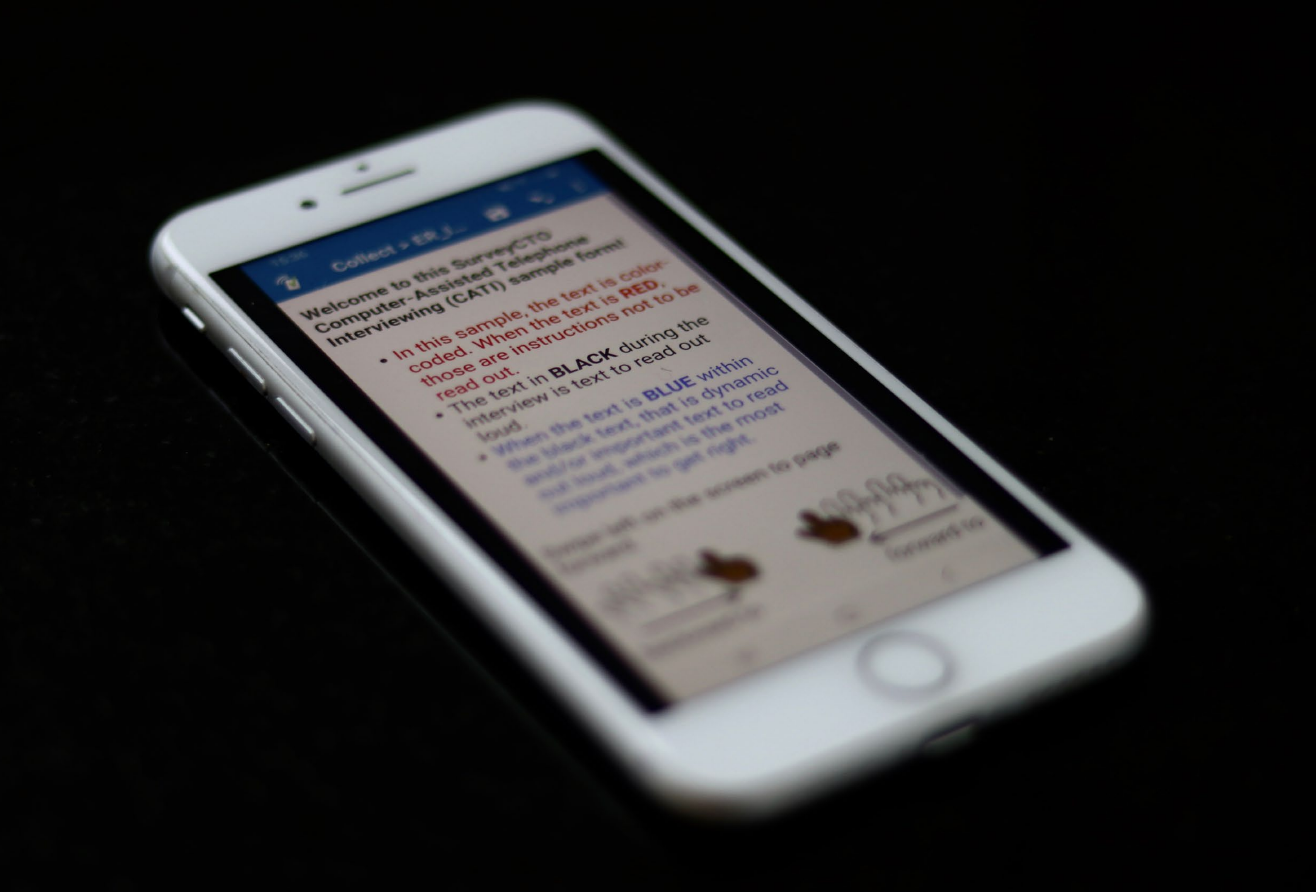

GOING DIGITAL

Computer-Assisted Telephone Interviewing (CATI): Lessons learned from a pilot study

www.oxfam.org

OXFAM

In this sixth instalment of the Going Digital Series, we share our experiences of using computer-assisted telephone interviewing (CATI) software, which was researched and piloted following the outbreak of COVID-19 and the subsequent need for improved remote data collection practices.

© Oxfam International May 2021

This case study was written by Melissa Harris and Alexia Pretari. Oxfam acknowledges the assistance of Ric Tighe, Melton Llenos, Amy O’Donnell, Jaynie Vonk, Hannah Fisher-Jones, Helen Wishart and Julia Bruce in its production. The pilot was conducted in collaboration with Oxfam in Iraq and the Iraqi Al Amal Association: Sabah Ali, Zhean Ismael, Mustafa Tuaib, Mohammed Fadhel and Surood MF Ahmad. The pilot was made possible through collaboration with Optimum Analysis and specifically Sarah Nijholt, Mahran Alhaeyk, Farah Abdulrazzaq Salih, Kayghan Muhamed and Saeed Taher. Oxfam would also like to thank SurveyCTO for their contributions, specifically Amrik Cooper, Marta Costa and Max S. Haberman. This study is part of a series of papers written to inform public debate on development and humanitarian policy issues.

For further information on the issues raised in this paper please email Melissa Harris (mharris1@oxfam.org.uk).

This publication is copyright but the text may be used free of charge for the purposes of advocacy, campaigning, education and research, provided that the source is acknowledged in full. The copyright holder requests that all such use be registered with them for impact assessment purposes. For copying in any other circumstances, or for re-use in other publications, or for translation or adaptation, permission must be secured and a fee may be charged. Email policyandpractice@oxfam.org.uk.

The information in this publication is correct at the time of going to press.

Published by Oxfam GB for Oxfam International under ISBN 978-1-78748-758-1 in May 2021. DOI: 10.21201/2021.7581
Oxfam GB, Oxfam House, John Smith Drive, Cowley, Oxford, OX4 2JY, UK.

Cover photo: The SurveyCTO CATI format onscreen. Credit: Abbie Chilton.

1 INTRODUCTION

Since 2015, Oxfam GB has been documenting its transition from paper to digital data collection, and the benefits and considerations connected to this, through its Going Digital Series.[1] In this paper, we will introduce computer-assisted telephone interviewing (CATI) software, which was researched and piloted following the outbreak of COVID-19 and the subsequent need for improved remote data-collection practices. CATI is a surveying technique where interviews are carried out via a phone call, using an electronic device to follow a survey script and enter the information collected. A variety of software is available and we have been using SurveyCTO's CATI Starter Kit.[2] SurveyCTO was selected as one of Oxfam's standard tools because it meets wider data protection and privacy standards, as discussed in the first and fourth issues of the *Going Digital* series (Tomkys Valteri and Lombardini, 2015; Vonk, 2019). In section 2 of this paper, we will present an overview of what CATI is, as well as how its use changes the whole phone interview process.

We will then discuss and reflect on the advantages as well as the challenges and limitations we encountered when piloting this during an impact evaluation in Iraq. The pilot took place as part of Oxfam GB's Effectiveness Review series,[3] in which Oxfam and its partnering consultant, Optimum Analysis, conducted phone interviews with women affected by conflicts in Kirkuk Governorate. Two researchers conducted 44 interviews in October 2020, asking both open-ended and closed-ended questions, generating a mix of textual and pre-coded data.[4] While the technology enables the digitalization of phone interviews, it is only one aspect of carrying out such interviews. The technology can enhance or strengthen certain aspects, such as data protection. On the other hand, additional practices need to be put in place alongside it to minimize the risk of doing harm. This is the case when considering privacy more broadly, for example. For this reason, in section 3, we will consider the use of the technology in the context of the whole interview protocol and will reflect on three themes that appeared critical throughout the pilot:

1. Data protection and privacy;
2. Monitoring of the interview process; and
3. Data quality, including audio recordings.

The lessons shared will be relevant beyond the scope of the CATI software provider we used in this pilot.

The annexed CATI Resource Pack shares practical documentation: step-by-step set-up guidance, Excel templates, plug-ins, training material and an example interview protocol for using CATI within SurveyCTO.

2 WHAT IS CATI? WHAT IS CATI SOFTWARE?

Phone interviews are a familiar data-collection technique used within Oxfam and beyond and have generally been considered good practice when face-to-face data collection is not viable, for reasons such as access or security. The COVID-19 pandemic brought a renewed interest in such interviews, as face-to-face data collection carried a risk of transmission and became impossible in many contexts. Typically, conducting phone-call interviews involved using a laptop or tablet to enter data digitally or paper forms, while simultaneously launching calls from a mobile phone.

In its simplest form, computer-assisted telephone interviewing (CATI) is a surveying technique where interviews are carried out via a phone call with the interviewer using one electronic device (computer/smartphone/tablet) to both read the survey script and enter the information collected.

CATI software therefore uses technology to integrate these processes and, in turn, make them safer and more efficient and effective. While the breadth of features can differ, depending on the system and software being used, the main functionalities of CATI include:

- Digitalized survey scripts (including skip logic, constraints, etc.) and data entry, e.g. the interviewer reads questions from a screen and enters data directly into the same digital device (e.g. smartphone).
- In-built call functionalities: the interviewee is selected from a call list and then the call is launched from within the CATI software.
- Reporting and analytics are available in real time showing details such as response rate, average call duration, etc.

For comparison, Table 2.1 shows the process of conducting a phone interview using the CATI software, alongside the approach typically taken without it.

The resource pack annexed to this case study provides practical guidance on how to use SurveyCTO’s CATI Starter Kit. In Section 3 of this paper, we link to the relevant sections of the step-by-step guidelines, templates and materials of the resource pack, in the ‘In practice’ boxes.

Table 2.1: Conducting phone interviews with and without CATI software

	Process using SurveyCTO's CATI Starter Kit	Process NOT using CATI software
Survey design	Digital survey is prepared on SurveyCTO using the templates in the CATI Resource Pack.	Survey is prepared, using either one of Oxfam's approved mobile data collection tools or paper, e.g. SurveyCTO, Mobenzi or ODK.
Interviewee list	A 'call list' containing details (including phone numbers and unique IDs) of the interviewees is prepared as an MS Excel file and uploaded to the SurveyCTO server. Interviewees can be assigned to specific interviewers, to reduce access to personal information.	A call list containing personal information of the interviewees is prepared using either MS Excel or Word. This is shared with interviewers either digitally or as paper copies.
Selecting an interviewee	The interviewer opens the software on their mobile phone and selects a person on their personalized digital call list. They can only see cases that have been assigned to them.	The interviewer selects a person on the call list. The interviewer will be able to see details of all cases (not just those to which they are assigned) unless personalized call lists have been made for each interviewer.
Starting the interview	Once the interviewee is selected a phone dialler appears within the software on the mobile device, automatically displaying the phone number of the selected interviewee (numbers can also be masked – see section 4). The interviewer can then press a button to begin the call.<br>To start the interview, the interviewer moves to the next screen and is presented with the interview script. The interviewee's answers can then be entered into the device using either the touch screen or keypad.<br>The interviewee's ID is **automatically** linked to the call and subsequent generated data.<br>If the interviewee wishes to postpone the call to another time, this can be captured by the interviewer within the survey script. The call list will then be automatically updated to show the rescheduled call time.	The interviewer manually enters the interviewee's number into a phone and launches the call.<br>At the same time, the interviewer must open the survey script. This could be a paper script or appear on separate digital device, such as a laptop.<br>The interviewer **manually** selects or enters the interviewee's ID in the digital form or on the paper questionnaire.<br>If the interviewee wishes to postpone the call to another time, the interviewer will need to manually record this on the interviewee call list.
Ending the interview	The interviewer will either complete or reschedule the interview depending on the interviewee's preference.<br>Details of the call are **automatically** updated on the case list. For example, if a respondent does not answer, the call list will update to show a call attempt was made and automatically reschedule a second attempt to the following day.	The interviewer will either complete or reschedule the interview depending on the interviewee's preference.<br>Details of the call are **manually** updated by the interviewer on the call list.
Data transmission and storage	Before transmission to the server, the data generated through the interview is automatically encrypted on the device to maximize data security.[5] This would include any personal information from the interviewee list that was preloaded to the form (such as phone number and name) to identify the interviewee.<br>The interviewee list itself is stored on the server and on the devices, and access is restricted to specific users and password-protected.	Depending on the software used, the digital data generated could be automatically encrypted, but additional manual steps need to be put in place to protect digital interviewee lists.<br>For paper interviewee lists and/or paper surveys, additional steps need to be put in place (such as daily storage in a locked cabinet).

3 LESSONS LEARNED FROM A PILOT

3.1 DATA PROTECTION AND PRIVACY

Enhanced protection of personal data and encryption

One of the most significant benefits of using SurveyCTO's CATI Starter Kit is improved data protection and privacy. The software has several features that help to control and limit access to personal data of interviewees (names and phone numbers at least).

Typically, without the software, the contact details of respondents would be stored centrally in a document, e.g. MS Excel. Even if efforts are made to protect this document from those outside the research team, and to share it safely within the team (through Box[6] for example, which provides another layer of encryption) producing separate lists for each interviewer would be labour-intensive. In cases where separate lists are not created, one central list contains large quantities of personal data which can then be accessed by all the researchers, not only the person who will be calling a given individual.

By comparison, SurveyCTO's CATI Starter Kit allows the assignment of calls to each interviewer. Only one person needs to have access to the file containing all personal data and must upload it to the software (the administrator); interviewers will only be able to see the personal data details of the calls to which they have been assigned.[7] Furthermore, access to the call list requires a username and password, meaning the personal data is further protected. This digitalization of the call list, which remains available with or without internet access, also reduces the risk of leaving a paper trail.

Regarding the privacy of the interviewees, another notable feature of this software is the ability to constrain call attempts. This allows the research team to decide a maximum number of calls that can be made to each interviewee, after which the respondent's details will automatically disappear from the call list and no further call attempts can be made. This feature helps to protect interviewees from receiving high volumes of calls. During the pilot, the maximum call attempt was set at five. Since interviewees had already received a preliminary call[8] and consented to taking part in the interviews, this volume of calls was considered acceptable. However, in instances where prior consent had not been obtained, a maximum of three call attempts would be more appropriate and is usually considered standard practice.

It should also be noted that SurveyCTO was selected as one of Oxfam's standard tools because it meets wider data protection and privacy standards. 'In terms of data protection, our key requirements are (1) having servers hosted in the EU, (2) using encryption during transmission, and (3) encrypting data from finalized questionnaires on mobile devices' (Vonk, 2019). In the setting of CATI, the encryption feature of SurveyCTO enables the phone numbers and other personal data to be protected when the data is transmitted and stored, between the mobile devices and the server. Encryption is particularly important as once the interview is completed, the personal data will be automatically linked to novel information gathered through the interview, some of which may be sensitive. This is because some of the personal data is preloaded to the form in order for the interviewer to identify the interviewee.

The data will then be stored on the SurveyCTO server and only be accessible with the encryption key.

**In practice – relevant documents from the Resource Pack**
• Set Up Guidelines: Steps 2 and 7.3<br>• Material: Respondents Templates.xlsx

Privacy, safeguarding and protection protocols

Switching from face-to-face interviews to phone interviews also raises questions related to privacy during the interview process, safety and protection. While the technology can enhance or strengthen certain aspects, others require specific steps to be put in place alongside the technology to avoid causing harm.

First, we should highlight that considerations around privacy and safety depend on the power dynamics at play in a given context – who owns the phone, whether the phone number is likely to be shared between individuals, who is likely to pick up or use the phone, and how norms, especially gender and ageist norms, affect who can safely speak to a stranger on the phone alone. These considerations also depend on the set-up of the interviews: Who is interviewed? Who conducts the interview? What are the topics covered? On the latter, it is important to highlight that UN Women recommends not carrying out phone interviews to ask questions related to the prevalence of domestic violence, as the risk for the survivor is too high (see this decision tree about data collection on violence against women and COVID-19).[9]

In this section, we draw on the experience of the pilot, where we aimed to talk to women who had been displaced or who have been living in areas where people were displaced because of conflicts in the area. The interviews included questions related to their economic activities, as well as any history of displacement, hopes for the future and opinions related to trust in the community, gender norms or the acceptability of domestic violence. Difficult stories were likely to be shared and the set-up of the interviews was designed so they could happen safely, privately and in a responsible manner.

Interview protocol to maximize privacy and safety

One of the features that the technology enables is for interviewers to systematically conduct a pre-call to confirm with the interviewee that they are interested in taking part in the interview, and to set up a time and date that would work for the interviewee and where they could be alone. This was deemed particularly important in a context where a phone number can be shared between several household members, and we aimed to talk to women alone.

However, the pre-call does not entirely ensure privacy or safety. We took an adaptive approach, depending on the extent to which privacy could be ensured.

- First, at the beginning of the interview, the interviewer would always ask whether the interviewee is alone and record this information. If she was not, the interviewer would then check if it was possible to reschedule at a time when she would be alone.
- Second, while the ideal scenario was for the interviewee to be alone, if the interviewee had a strong preference to be with someone during the interview, the interviewer would go ahead and schedule the interview as wished. During the interview itself, the interviewer would skip the sensitive questions.

- Third, in a setting where a phone number can be shared with several household members, and men in particular are more likely to be the primary user of the phone, there could be situations where it is not possible at all to talk to the intended interviewee – for instance, the husband of the intended interviewee refusing to allow the interviewer to speak to her directly. In such cases, researchers decided that to interrupt the process abruptly and not carry out the interview at all was too risky for the intended interviewee, as it could bring suspicion. As a consequence, it was decided that in such instances, a very short version of the interview would be carried out (excluding all sensitive questions and more as appropriate), to avoid any suspicion around the interview and any risks for the intended interviewee. While this shortened version was not built into the survey during the pilot, for future data collection this could be easily integrated into the survey design using skip logic.
- Finally, the research team agreed on a safe word in advance, to stop the interview in case someone comes in while the interview is ongoing. At the beginning of the interview, the interviewer would introduce the safe word to the interviewee and let them know that should they use it, they will switch topic, stay on the phone for a few minutes and reschedule the interview.

All of these points were developed as part of the interview protocol, discussed during the interviewer training and adapted as needed based on how situations evolved.

These steps were taken to maximize privacy and safety. However, if the chances that the interviewee would not disclose whether someone was alongside them were considered too high, which could be more so the case in homes where there is domestic violence, the research team could decide to change the questions asked, or to not carry out the interview at all.

Reporting protocol for safeguarding and protection

Even when not prompted directly, difficult stories can be shared and these have to be handled responsibly. Clear reporting protocols need to be in place, in consultation with safeguarding and protection specialists, and have to be discussed in the interviewer training. The interview form is not a reporting mechanism and information related to safeguarding or protection should not be entered into the interview form, as this would neither be safe nor appropriate for safeguarding or protection staff to take action (as they would not have access to the data generated). For this reason, the appropriate reporting or feedback mechanisms have to be openly shared with interviewees. The technology itself can only marginally facilitate this process by integrating some key information in the script for interviewers to share.

Based on our experience and in collaboration with safeguarding and protection staff, we identified three key elements:

- Having a clear reporting protocol for cases of safeguarding.[10] The protocol will highlight the duty to report, even including anonymously, and the confidential channel to do so. It will also highlight the fact that the interviewer should not ask any questions related to the matter but ask permission to share the personal contact information of the interviewee.
- Having a clear reporting protocol for cases of protection (sexual exploitation, abuse, harassment or violence towards children and adults) in which the perpetrator is not a member of Oxfam staff or partner staff.
- Informing each and every interviewee of available feedback mechanisms, misconduct reporting channels and any other relevant referral pathways.

These three key elements should be developed in detailed and specific protocols and adapted to the context in which the interviews are taking place, by consulting the interviewers involved in gathering the data, as well as safeguarding and protection staff. Interviewer training will put emphasis on the fact that the interview form is not a reporting mechanism, and that stories related to safeguarding or protection should be reported confidentially following appropriate reporting mechanisms and processes. The training will also put emphasis on preparing interviewers to receive such stories, in case they are shared.[11]

**In practice – relevant documents from the Resource Pack**
• Material: Privacy and reporting protocol_CATI.doc

3.2 MONITORING THE DATA GATHERING PROCESS

Real-time data visualization

A key advantage of using SurveyCTO's CATI Starter Kit is the ease of integrating incoming information into a dashboard. A dashboard is simple to set up and data is updated in real time, providing live oversight of ongoing data collection. This can be used by interviewers and the administrator to monitor progress (e.g. the number of completed interviews) as well as quality (e.g. very long or short calls). Note that when using encryption, the fields that will be exported to the dashboard need to be marked as 'Publishable' in the main form.[12] This should only be done for fields that are not sensitive and do not contain any personal data.

Figure 3.1: Example of a monitoring dashboard

Cases by status	Column Labels					
User	Complete	No answer	Wrong numbe	Rescheduled	Refused	Grand Total
Researcher 1	6	10	12	8	1	37
Researcher 2	17	1	2			20
**Grand Total**	**23**	**11**	**14**	**8**	**1**	**57**

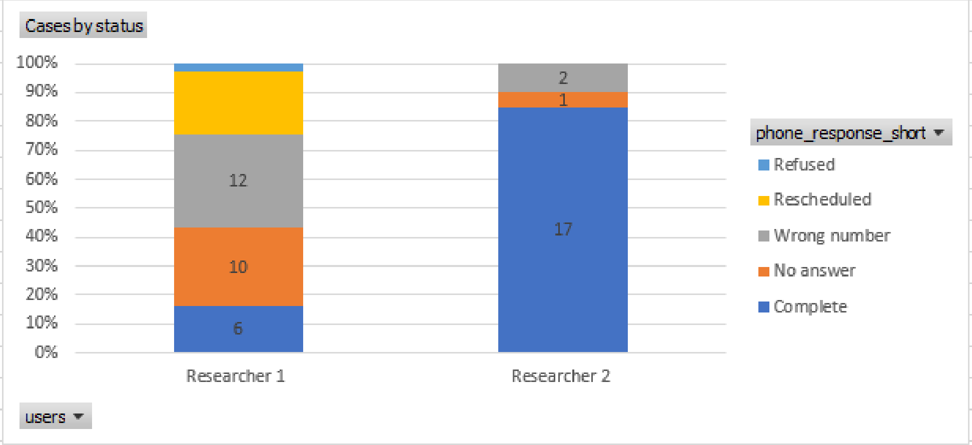

**In practice – relevant documents from the Resource Pack**
• Set Up Guidelines: Step 6

Interviewer and interviewee experience

SurveyCTO's CATI Starter Kit has several features that makes the process relatively quick and easy for interviewers.

Firstly, when opening the software, the interviewer is presented with a list of interviewees they have been assigned to call (see Figure 3.2). Interviewers also have the option to sort the call list by the 'Call back time' column, so they can view their upcoming calls chronologically, making the process easier to track.

Figure 3.2: Example of a call list

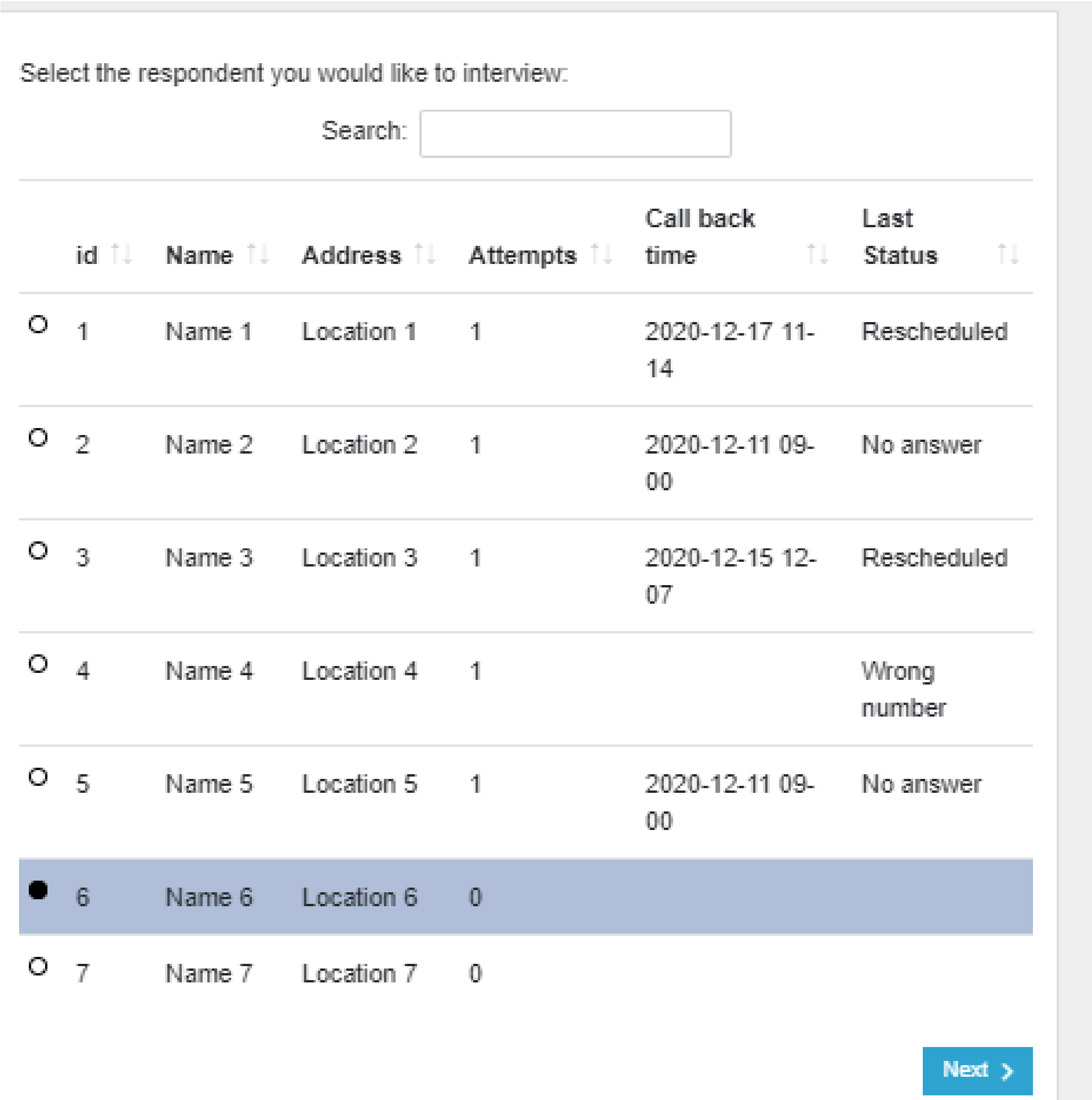

Select the respondent you would like to interview:

Search:

	id	Name	Address	Attempts	Call back time	Last Status
○	1	Name 1	Location 1	1	2020-12-17 11-14	Rescheduled
○	2	Name 2	Location 2	1	2020-12-11 09-00	No answer
○	3	Name 3	Location 3	1	2020-12-15 12-07	Rescheduled
○	4	Name 4	Location 4	1		Wrong number
○	5	Name 5	Location 5	1	2020-12-11 09-00	No answer
●	6	Name 6	Location 6	0		
○	7	Name 7	Location 7	0		

Next

When launching the call, the interviewer does not need to manually enter the telephone number into the mobile phone, as it will be automatically populated in the dialler once an interviewee is selected. This saves time and limits the chances of a mistake. If the call drops at any point during the interview, which can occur frequently in locations with poor network connectivity, it is possible to call back without manually entering the number.

This 'call list' will automatically update depending on the outcome of a call, reducing the burden on interviewers to document and track the status of each call. When a call has been

completed, or the maximum number of call attempts has been reached, the number will be automatically removed from the call list (see Figure 3.3). When using encryption, the fields in the survey form related to the outcome of the call need to be marked as publishable for the data to go through to the interviewee call list and be automatically updated.

A note on consent

When referring to consent in the diagram overleaf, we are referring to informed consent, and recognize that power dynamics and other factors play an important role within this. You can find more information on informed consent from one of our earlier papers in this series: Going Digital: Privacy and data security under GDPR for impact evaluation (Vonk, 2019).

Figure 3.3: Workflow of the call list

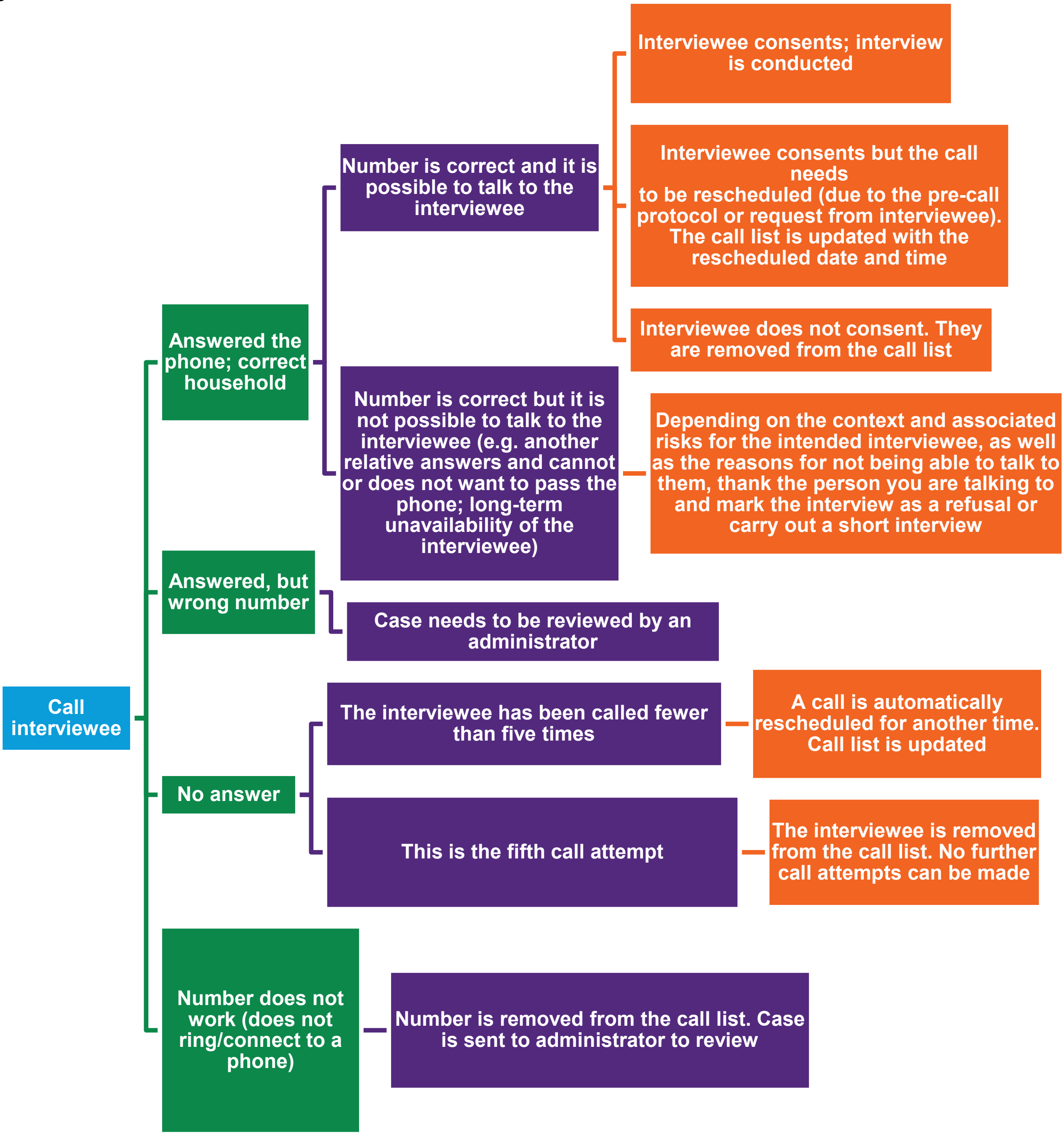

This automatic update to the interviewee call list is achieved through closely integrating the phone interview process into the survey script. Figure 3.4 shows the questions in the script

that recreate the logic presented in the flow chart above. For the interviewer, this is also helpful in guiding the interview process – rather than immediately opening with the survey questions, this integration contextualizes the script to a phone interview, recognizing that this process is different from face-to-face interviews and there are different paths the call could take, e.g. the phone number being incorrect or someone else from the same household picking up the phone. By considering these pathways within the script, it also ensures standardization of processes, guaranteeing each interviewer is handling each call in the same way. While such integration makes it easier, interviewer training and collective fine-tuning of such pathways and scripts is also critical, as well as communication throughout the data-gathering exercise to ensure context-specificity and flexibility.

Following the pilot, this call list functionality was cited by the data-collection team as one of most significant advantages of using the CATI software.

Figure 3.4: Integration of phone call process into survey script

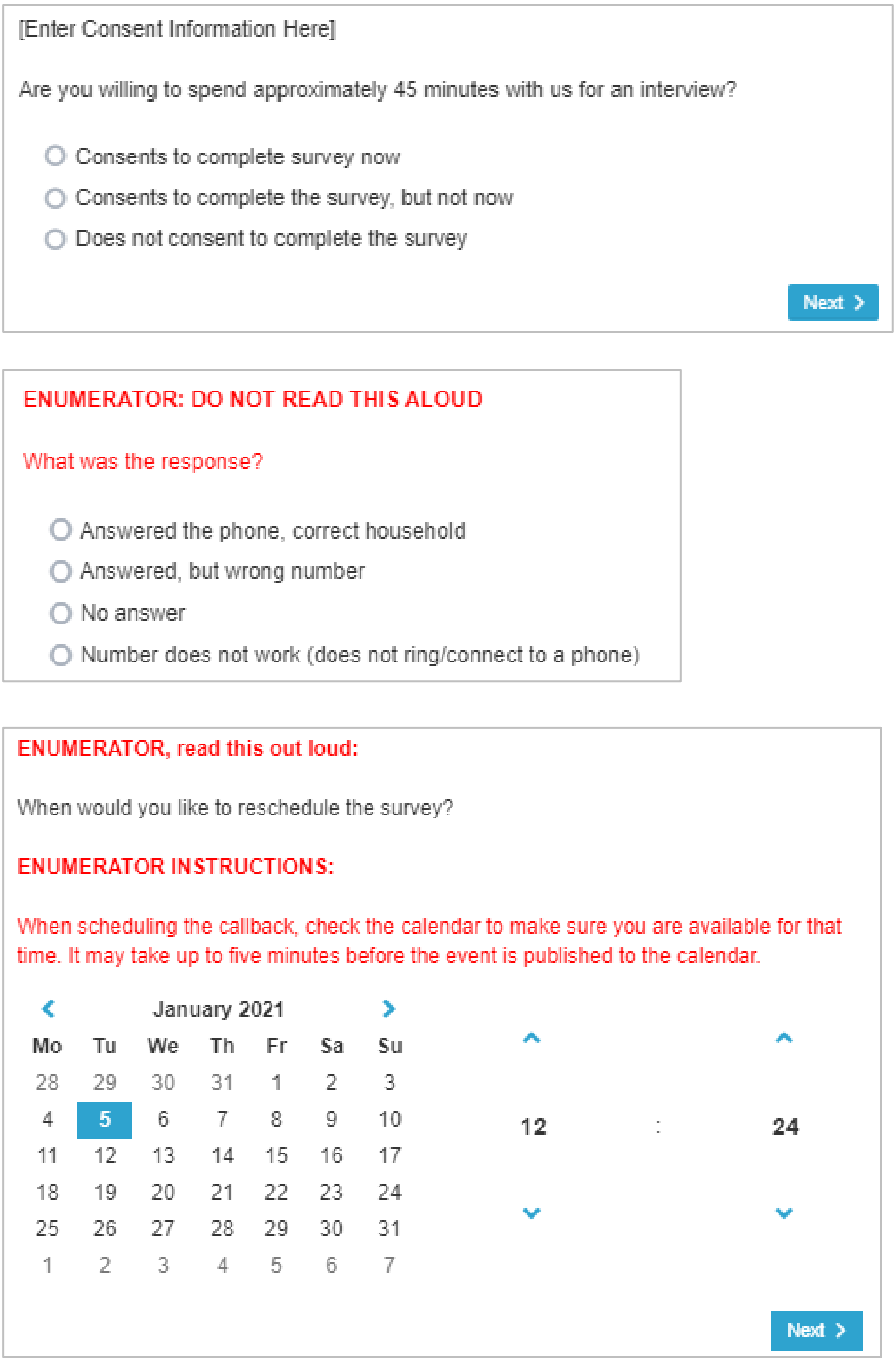

While all the features above improve the process of data collection for the interviewer, the aim is also to make the phone interview process safer and more enjoyable, comfortable and convenient for the interviewee as well. For example, by making the process to reschedule calls more straightforward, it is more likely interviewees will feel comfortable with taking this option if that is what they prefer or it is more convenient for their schedule, as the automation means it is easy for the interviewer to reschedule the call. It also makes it easier to systematically do a pre-call dedicated to setting up a convenient time for the interview, which we believe is a simple way to improve privacy and safety, and to give a better experience to the interviewee (as mentioned in section 3.1).

**In practice – relevant documents from the Resource Pack**
• Set Up Guidelines: Steps 1 to 4<br>• Material: CATI Survey Template.xlsx; Respondents Template.xlsx

3.3 DATA QUALITY AND AUDIO RECORDINGS

Audio recordings

One of the features provided by many CATI software packages is call recording, which, if used safely, can be one of the most effective ways to monitor the performance of interviewers and identify issues that were not foreseen during interviewer training. This feature is especially useful within the context of the COVID-19 pandemic, where interviewers are often working remotely.

In the pilot, the option to record calls was particularly appealing given that the interview was largely qualitative. As well as providing monitoring insights, the recordings would be useful to retrospectively add to the interviewer's notes, as well as support the translation process and subsequent analysis. Further, SurveyCTO's audio recording functionality (known as audio audit[13]) has been designed with safety at the forefront. In addition to secure storage of the recordings,[14] it was useful that recording could be activated through skip logic, and therefore only after consent for recording had been obtained. The duration and frequency of its use can also be tailored to each survey, meaning that recordings are not captured unnecessarily or when potentially sensitive information could be shared.

However, despite the intention to use the audio audit feature, one challenge that arose during the testing stage of the pilot was that the functionality only works on devices that run on Android 4.4.8.[15] In addition, in most instances the feature only allows for recording on the interviewer's side; while it is possible to hear what the interviewer is saying, the responses from the interviewee are not captured in the recording. While this is adequate for monitoring purposes, it meant that the recordings could not be used to supplement interviewer's notes as intended.

Despite efforts to procure suitable devices locally that met this specification, they were not available, and while there are alternative options for audio recording, such as using the Twilio Field plug-in,[16] these were not possible to consider because of time constraints.

In practice – relevant documents from the Resource Pack
• Set Up Guidelines: Step 7.1

Capturing textual qualitative data

While many of the advantages of using CATI software stem from its centralization of data, whereby both the phone call and survey are launched from the same software and device, this also created challenges for the collection of qualitative data during the pilot.

As mentioned earlier, when conducting phone interviews without CATI software, it was common to launch the phone call while simultaneously entering data into a webform via a laptop. For data entry, this is easy and comfortable for the interviewer; webforms can be navigated using a mouse and typing longer qualitative responses is generally quicker on a laptop than a phone or tablet, particularly if the interviewer is wearing a headset. However, when using SurveyCTO's CATI Starter Kit, the device used for data entry must also have the capability to launch phone calls. While some tablets do have this functionality, in most cases, and in the case of the pilot, smart phones are likely to be more available and affordable.

During the testing stage of the pilot, it became apparent that it would not be feasible for the qualitative answers to be directly entered in the smartphone; the process was too slow for writing detailed responses and took too much of the interviewee's time. As a solution, it was decided that while quantitative data would be directly entered into the mobile phone, for the qualitative responses interviewers would handwrite short notes on paper (with unique ID and without personal data). As soon as the interview was completed and the form submitted, the notes were typed[17] and completed, reviewed, further anonymized and edited in an Excel file for translation and analysis.

For future data collection, Bluetooth keyboards could be explored as a possible solution to these challenges. These can be connected to smart phones as well as tablets and could therefore help to capture textual qualitative data quickly while using CATI. However, this would result in additional costs and may not be feasible in many circumstances.

General features for monitoring quality

One of the challenges previously faced with typical phone interviews has been the limitations in checking and monitoring interviewers' progress, primarily because of the decentralization of data. By launching the phone call from a separate device to the one being used to digitally enter the data, the usual monitoring checks, such as checking survey duration, become unreliable. In the absence of CATI software, while a survey duration can be calculated based on the time the interviewer took to fill in the digital survey form, the duration of the phone call, or tracking of the phone call attempts, is not available unless manually cross-checked with the phone log of the device used to make the call. These are easily automated with SurveyCTO's CATI Starter Kit, using functions like 'phone-call-duration()', for example. As well as the ability to see the outcome of each call when exported, submissions include a phone call log, containing the overall duration of the phone call, and markers signifying the time when the phone number was dialled, the time it was answered, and when it was ended.

You can find more information on monitoring quality with digital data collection from one of our earlier papers in this series: Going Digital: Improving data quality with digital data collection (Lombardini, Pretari & Tomkys Valteri, 2018).

In practice – relevant documents from the Resource Pack
• Set Up Guidelines: Steps 1 to 3<br>• Material: CATI Survey Template.xlsx

4 CLOSING THOUGHTS

Throughout the pilot, the use of the CATI software was shown to bring improvements in numerous areas of the data-gathering process. Improved data protection and privacy were a key advantage. Centralization of the data allowed for easier and improved data monitoring, as well as making the process more comfortable and convenient for both the interviewee and interviewer. Where SurveyCTO subscriptions have already been purchased, the CATI functionalities come at no extra cost, meaning it is a solution that can be adopted widely by INGOs such as Oxfam, as well as their partner organizations.

The pilot did, however, also identify some limitations of the software. Challenges around capturing qualitative textual data mean that the software may not be adequate or suitable for certain kinds of data collection. In addition, restrictions around audio recordings meant that it was not possible to use them to support data analysis and translations, as well as to conduct planned monitoring checks. While both of these issues could be overcome by purchasing specific hardware, budget constraints are likely to make this unfeasible in many circumstances.

Going forward, we are excited to explore additional features within SurveyCTO's CATI Starter Kit. The integration of the cloud communication platform, Twilio, which SurveyCTO is currently piloting, could resolve the challenges faced around using the audio audit functionality. It would indeed allow calls to be recorded regardless of the device specification, which would be really convenient as long as the data storage features of the cloud meet GDPR and Oxfam's privacy requirements and allow for confidential storage. In addition, the call masking feature means that even those who are launching the calls are unable to see the phone numbers of interviewees. Depending on the context, this feature could contribute to enhancing privacy and safety. Understanding the social context in which the calls take place, for both the interviewers and interviewees, will lead to understanding risks and to making sure that CATI is an enabler, rather than a tool that further entrenches social inequalities or causes harm.

REFERENCES

Hastie, R. and O’Donnell, A. (2017). *Responsible Data Management training pack*. Oxford: Oxfam GB. https://policy-practice.oxfam.org/resources/responsible-data-management-training-pack-620235/

Lombardini, S., Pretari, A. and Tomkys Valteri, E. (2018). *Going Digital: Improving data quality with digital data collection*. https://policy-practice.oxfam.org/resources/going-digital-improving-data-quality-with-digital-data-collection-620522/

Lombardini, S. and Tomkys Valteri, E. (2017*). Going Digital: Using and sharing real-time data during fieldwork*. Oxford: Oxfam GB. https://policy-practice.oxfam.org/resources/going-digital-using-and-sharing-real-time-data-during-fieldwork-620432/

Schwitter, N. and Liebe, U. (2020). *Going Digital: Web data collection using Twitter as an example*. Oxford: Oxfam GB. https://policy-practice.oxfam.org/resources/going-digital-web-data-collection-using-twitter-as-an-example-620948/

Tomkys Valteri, E. and Lombardini, S. (2015). *Going Digital: Using digital technology to conduct Oxfam’s Effectiveness Reviews*. Oxford: Oxfam GB. https://policy-practice.oxfam.org/resources/going-digital-using-digital-technology-to-conduct-oxfams-effectiveness-reviews-578816/

Vonk, J. (2019). *Going Digital: Privacy and data security under GDPR for quantitative impact evaluation.* Oxford: Oxfam GB. https://policy-practice.oxfam.org/resources/going-digital-privacy-and-data-security-under-gdpr-for-quantitative-impact-eval-620884/

NOTES

1 Going Digital: Using digital technology to conduct Oxfam's Effectiveness Reviews pilots (Tomkys and Lombardini, 2015); Going Digital: Using and sharing real-time data during fieldwork (Lombardini and Tomkys Valteri, 2017); Going Digital: Improving data quality with digital data collection (Lombardini, Pretari and Tomkys Valteri, 2018); Going Digital: Privacy and data security under GDPR for quantitative impact evaluation (Vonk, 2019); and Going Digital: Web data collection using Twitter as an example (Schwitter and Liebe, 2020).

2 Costa, M. (2021, March 5). *Computer-assisted telephone interviewing (CATI) starter kit.* SurveyCTO Support Center. https://support.surveycto.com/hc/en-us/articles/360044958494-Computer-assisted-telephone-interviewing-CATI-starter-kit

3 Search results on https://policy-practice.oxfam.org/ for 'Effectiveness Review Impact Evaluation'. https://policy-practice.oxfam.org/?tab=all&s=Effectiveness+Review+Impact+Evaluation#filter-results

4 The full report will be accessible on the Policy and Practice website once finalized: https://policy-practice.oxfam.org/

5 It should be noted that currently the call list server datasets are not encrypted on the device, however risks are mitigated through SurveyCTO's private app storage and admin settings (see the Resource Pack). As part of being compliant with GDPR, we see encryption as one recommended feature to reduce risk when handling personal data (although not a required one), as we discussed in *Going Digital 4*: https://oxfamilibrary.openrepository.com/bitstream/handle/10546/620884/cs-going-digital-gdpr-181019-en.pdf?sequence=1

6 Box website: https://www.box.com/en-gb/home

7 It should be noted that the full call list is downloaded onto the device but is subsequently filtered through the 'users' column.

8 Oxfam's partner organization pre-contacted interviewees, to build on the trustful relationship they had with them, as well as to ensure explicit consent was given for personal information to be shared with the consultancy team in charge of conducting the interviews.

9 UN Women (n.d.). *Data Collection on Violence Against Women and COVID-19: Decision Tree*. PowerPoint presentation. https://www.unwomen.org/-/media/headquarters/attachments/sections/library/publications/2020/decision-tree-data-collection-on-violence-against-women-and-covid-19-en.pdf?la=en&vs=3346

10 'Safeguarding in Oxfam is a set of procedures, measures and practices to ensure that Oxfam upholds its commitment to prevent, respond to, and protect individuals from harm committed by staff and related personnel. In Oxfam, we focus on Sexual Exploitation, Sexual Abuse, Sexual Harassment (SEAH) and child abuse. We do this by: (i) listening to those who are affected; (ii) responding sensitively and safely when harm or allegations of harm occur; and (iii) learning from every case.' One Oxfam Safeguarding Core Standards. See https://www.digna.ca/wp-content/uploads/2020/11/EN-One-Oxfam-Safeguarding-Core-Standards-quick-reference-May-2020.pdf.

11 Oxfam internal guidelines on MEAL and Safeguarding can be used to develop appropriate training materials.

12 'Publishable' here is a term used by SurveyCTO and is a component of form building and design.

13 SurveyCTO Documentation. Designing forms – core concepts. *Field type: audio audit*. https://docs.surveycto.com/02-designing-forms/01-core-concepts/03ze.field-types-audio-audit.html

14 Recordings are encrypted and are stored on the server and can only be accessed by the administrator using a private key.

15 SurveyCTO provides more guidance on device compatibility here: https://support.surveycto.com/hc/en-us/articles/360051630354-Guide-to-phone-survey-data-quality-control

16 Haberman, M. S. (2020, October 1). *Using Twilio with SurveyCTO to securely record phone calls only with consent.* https://support.surveycto.com/hc/en-us/articles/360055415333-Using-Twilio-with-SurveyCTO-to-securely-record-phone-calls-only-with-consent

17 This step can be avoided if the interviewers type their notes directly into an Excel file on a computer while the interview is ongoing. It depends on whether the equipment is available during the interview, and how comfortable interviewers feel with typing while listening.

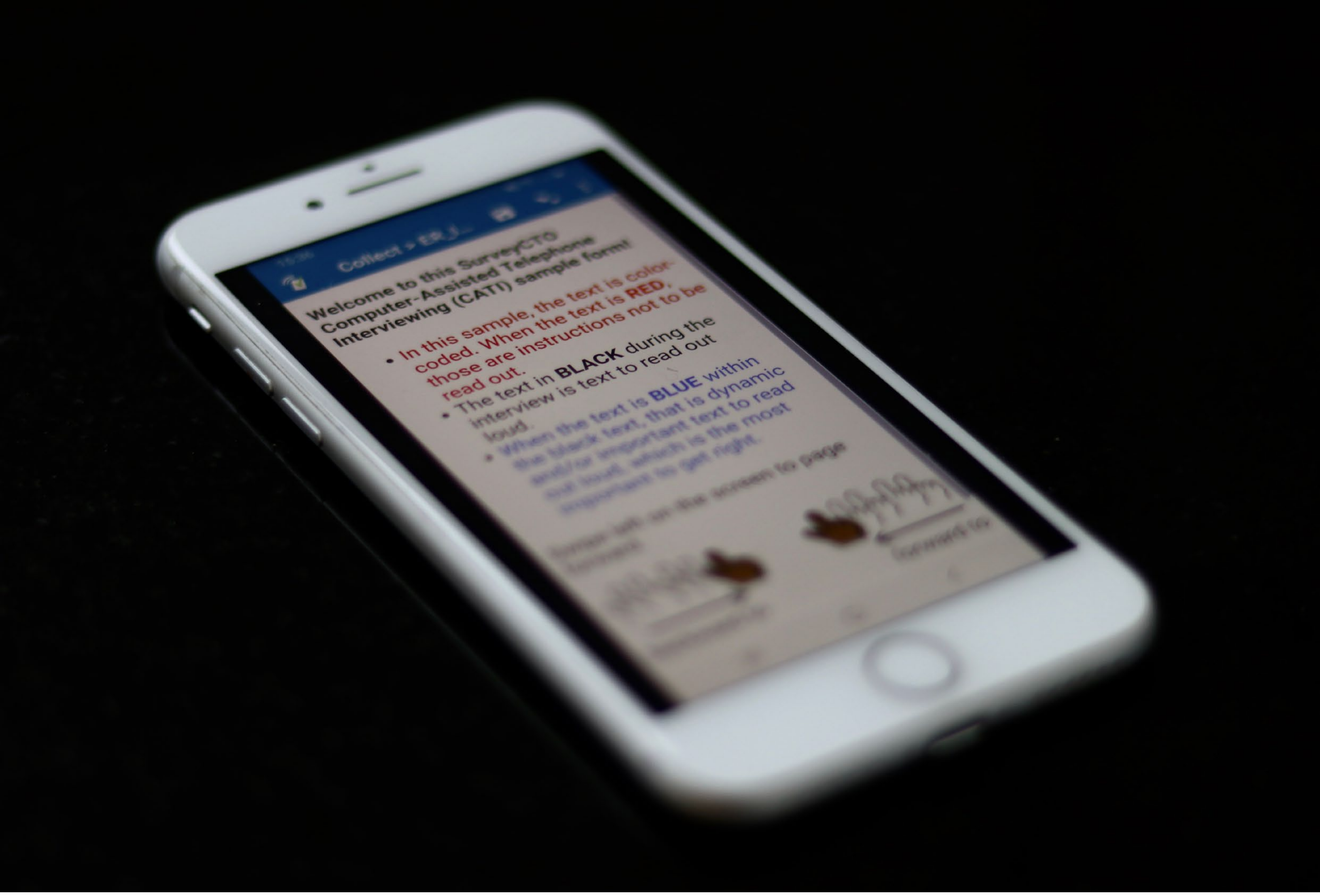

OXFAM

Oxfam is an international confederation of 21 organizations networked together in 67 countries, as part of a global movement for change, to build a future free from the injustice of poverty. Please write to any of the agencies for further information, or visit www.oxfam.org

www.oxfam.org
