# Supplementary material for: Public knowledge, practices, and awareness of antibiotics and antibiotic resistance in Myanmar: The first national mobile phone panel survey
Source: PLoS One. 2022 Aug 18;17(8):e0273380. doi: 10.1371/journal.pone.0273380 (PMC9387831; doi:10.1371/journal.pone.0273380)
Supplement: Attachment — Submitted filename: EN-One-Oxfam-Safeguarding-Core-Standards-May-2020.pdf [file pone.0273380.s005.pdf]

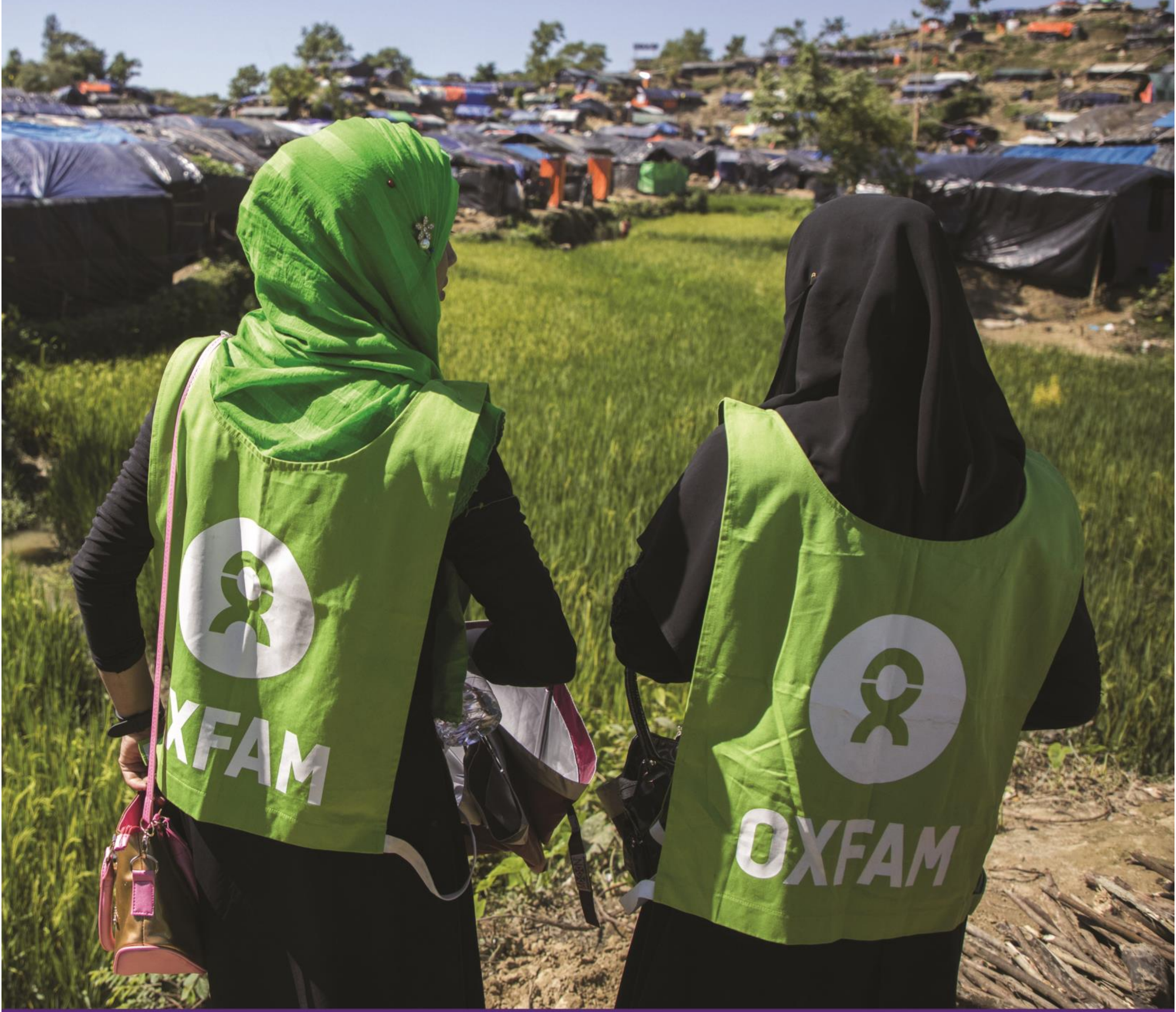

ONE OXFAM SAFEGUARDING CORE STANDARDS

May 2020

and guidance for implementation

Produced by the Safeguarding Shared Service

Image source: Saikat Mojumder (Oxfam Novib)

OXFAM

INTRODUCTION

Welcome to the One Oxfam Safeguarding Core Standards.

These standards provide working guidance on how to implement the One Oxfam Safeguarding Policies.

Click Here

One Oxfam PSEA Policy
One Oxfam Child Safeguarding Policy
One Oxfam Youth Safeguarding Policy
One Oxfam Digital Safeguarding Policy

Safeguarding in Oxfam is a set of procedures, measures and practices to ensure that Oxfam upholds its commitment to prevent, respond to, and protect individuals from harm committed by staff and related personnel. In Oxfam, we focus on Sexual Exploitation, Sexual Abuse, Sexual Harassment (SEAH) and child abuse. We do this by: (i) listening to those who are affected; (ii) responding sensitively and safely when harm or allegations of harm occur; and (iii) learning from every case.

Protection from Sexual Exploitation and Abuse (PSEA) refers to protecting vulnerable populations and communities from sexual exploitation and abuse by staff, partners and volunteers or as a direct result of Oxfam activities. *See also* PSEA Policy

Child Safeguarding refers to the policies, procedures and practices employed to safeguard children who come into contact with Oxfam and all those associated with the delivery of our work from all forms of harm, abuse or exploitation and the responsibility of all personnel to embed these at the activity level to ensure Oxfam is a child safe organisation. *See also* Child Safeguarding Policy

WHAT ARE THE ONE OXFAM CORE STANDARDS?

One Oxfam Safeguarding Core Standards summarise the commitments and requirements outlined in One Oxfam Safeguarding Policies and other strategic documents[1]. They outline the minimum requirements that Oxfam teams will work towards to establish an effective safeguarding system[2] to fulfil our internal and external obligations on Safeguarding, e.g. towards communities, donors and supporters. The Core Standards support Oxfam's ambition of transforming organisational culture, to model our values as an organisation and to prevent abuses of power that manifests as sexual exploitation and abuse and child abuse .

One Oxfam Safeguarding Core Standards are developed in line with the Minimum Operating Standards – Protection from Sexual Exploitation and Abuse by own Personnel (MOS-PSEA) developed by the Inter-Agency Standing Committee (IASC). These international standards form the basis of most donor policies on safeguarding.

[1] For example the One Oxfam Standard Operating Procedure for Case Management.
[2] The overall structure within a country/affiliate that contributes to prevention, detection and response of all issues relating to sexual exploitation & abuse and child abuse committed by any Oxfam representative. This involves organising the different aspects of safeguarding, including policy, procedures and practices, to ensure that they connect and work together to form one comprehensive system.

Although each core standard may look like a stand-alone area of focus, in effect they are inter-dependent. Teams need to work towards each standard at the same time to ensure systematic implementation of safeguarding measures. This will strengthen work across the continuum of prevention, detection and response.

WHO DO THE SAFEGUARDING CORE STANDARDS APPLY TO?

The One Oxfam Safeguarding Core Standards apply to all Oxfam teams-at Country, Regional, Affiliate and Oxfam International level. They maintain a cross functional focus, highlighting the importance of different functions in the organisation to ensure good safeguarding practices across Oxfam's work such as; management, safeguarding, programming, human resources, MEL, IT, media and communications.

Where standards apply to individuals representing Oxfam, this document uses the term employees and related personnel. This term includes all employees of Oxfam International, Oxfam Affiliates and Oxfam Country Offices. The term also includes board members, volunteers, interns, consultants, casual workers, in addition to individual and corporate contractors of these entities and related personnel. For the purpose of this document, this term denotes anyone involved in the delivery of Oxfam's work, eg. employees and related personnel of any partner organisations.

Leadership personnel are accountable for ensuring that their teams and programs meet these standards through proactive leadership and allocating sufficient human and financial resources. Oxfam teams are not expected to immediately comply fully with these standards; however, leadership personnel will work with their teams to put in place time- bound strategies to meet all core standards.

HOW DO YOU USE THIS DOCUMENT?

This document provides practical guidance for the implementation of each One Oxfam Safeguarding Core Standard. It supports Oxfam leadership and teams (global, affiliate, regional and country level) in understanding what is expected from them in relation to implementing the Core Standards and in strengthening their safeguarding system.

The document offers a comprehensive framework that outlines specific requirements broken down into minimum obligations and good practice guidance. It also describes how you need to demonstrate that you are effectively implementing each standard. It assists Oxfam teams to conduct an objective self-assessment of their safeguarding systems and practices. Teams can also use this framework to strategically plan and monitor safeguarding activities.

ONE OXFAM CORE STANDARDS AT A GLANCE

PRINT

Page 5 gives a one page infographic which provides the One Oxfam Core Standards "at a glance". **This can be used as a standalone poster/infographic** to be printed and displayed as required. This must also be used with the more detailed guidance found within this document.

SAFEGUARDING, SAFE PROGRAMMING AND PROTECTION

These are different but mutually reinforcing areas of work. This document focusses on safeguarding, but it is important to understand how these areas connect.

SAFE PROGRAMMING – "SAFE TO PARTICIPATE"

All people affected by our programmes can participate safely.
Our programmes do not:

- Cause harm to people; nor
- Undermine the values, standards and norms that underpin our work

We actively listen to communities, partners and staff. We analyse, monitor and manage all risks

Safe programming includes safeguarding actions to prevent or mitigate SEA or child safeguarding risks

Protection staff often provide technical support on safe programming. Protection programmes must use a safe programming approach

SAFEGUARDING

"SAFE FROM US"

Safeguarding in Oxfam is a set of procedures, measures and practices to ensure that Oxfam upholds its commitment to prevent, respond to, and protect individuals from harm committed by staff and related personnel.
In Oxfam, we focus on Sexual Exploitation, Sexual Abuse, Sexual Harassment (SEAH) and child abuse.

PROTECTION

"SAFE FROM OTHERS"

Dedicated staff working on programmes to improve the safety of civilians in conflict and crisis situations. Actions include:

- facilitating referrals for survivors of violence and abuse to emergency and protection services;
- community-based protection; and
- advocacy and campaigning.

ONE OXFAM SAFEGUARDING CORE STANDARDS

Safeguarding in Oxfam is a set of procedures, measures and practices to ensure that Oxfam upholds its commitment to prevent, respond to, and protect individuals from harm committed by staff and related personnel. In Oxfam, we focus on Sexual Exploitation, Sexual Abuse, Sexual Harassment (SEAH) and child abuse. We do this by: (i) listening to those who are affected; (ii) responding sensitively and safely when harm or allegations of harm occur; and (iii) learning from every case.

1. **A Safeguarding Plan is established:** A cross-functional Safeguarding Plan is developed for the implementation of One Oxfam Safeguarding Policies. Progress will be regularly monitored by management, and the plan updated annually or as required

2. **Trained Safeguarding Focal Points are present in all Oxfam offices:** All Oxfam teams identify and empower Safeguarding Focal point(s) to support the implementation of One Oxfam Safeguarding Policies. This will be proportionate to the scale of operations and safeguarding risk assessment.

3. **The Oxfam Code of Conduct is signed by all employees and related personnel:** All Oxfam employees and related personnel read, understand, sign and abide by the relevant Oxfam Code of Conduct.

4. **Safe recruitment and screening processes are in place:** Preventive measures are built into recruitment and screening processes to strengthen safeguarding.

5. **Regular and appropriate Safeguarding training is provided to all employees and related personnel:** Safeguarding training is tailored to the local context and to specific roles and functions and is provided at regular intervals.

6. **Safeguarding reporting and responding procedures are in place in all offices:** Contextualised, clear, survivor-centred procedures are in place for reporting and responding to safeguarding concerns.

7. **Community based feedback and complaint mechanisms are established:** Safe, confidential and accessible feedback and complaint mechanisms are established for raising safeguarding concerns and are always functional and well publicised; personnel and communities are aware and know how to use them.

8. **Partners adhere to One Oxfam Safeguarding Policies and Safeguarding Core Standards:** Oxfam's Safeguarding policies and core standards are clearly communicated to partners; Partners are supported to develop and/or implement their own Safeguarding policies and procedures.

9. **Safeguarding risk assessment and management practices are established:** Safeguarding risk assessment and management practices are in place for all Oxfam offices, projects and activities. Safeguarding risk assessments are undertaken for projects at the design phase and updated at regular intervals.

10. **Procedures for handling images and personal information are established:** Procedures are in place while collecting, storing and using images and personal information relating to communities as part of Oxfam's work.

OXFAM

1. A Safeguarding Plan is established

A cross-functional Safeguarding Plan is developed for the implementation of One Oxfam Safeguarding Policies. Progress will be regularly monitored by management, and the plan updated annually.

Why this standard is important?

Having an annual plan on safeguarding is essential to secure necessary resources to mainstream safeguarding across functions, operations and programs; ensuring leadership of management and monitoring. The plan supports Oxfam teams to create a roadmap to track systematic progress on all aspects of safeguarding and to incrementally achieve compliance to the One Oxfam Safeguarding Core Standards, through establishing priorities and milestones.

Putting the standard into practice (minimum requirements):

- ☑ Assess current safeguarding risks and gaps across different functional areas (eg. HR, Programs etc) and identify realistic measures under each standard to address these. Prioritise based on relevant criteria – eg. high safeguarding risks, implementation deadlines from donors etc.
- ☑ The safeguarding plan presents the activities that have a secured budget, identify the responsible personnel or function for their delivery, set deadlines for completion and outline the technical support that is needed from the organisation.
- ☑ The Safeguarding Focal Point or other appropriate person coordinates with all functions in the office to identify gaps and priorities to consolidate a draft plan for leadership review and approval. Technical support is sourced from Regional Safeguarding Advisors and Affiliate Safeguarding Leads as needed.
- ☑ To ensure adequate resourcing for the annual safeguarding plan, include 1-2% additional budget in all project proposals to cover safeguarding activities, where relevant.
- ☑ Leadership teams will review progress against the safeguarding plan at regular intervals, at least once every six months.
- ☑ Develop six-monthly progress reports to document work undertaken under each standard, key achievements, good practices and implementation challenges.
- ☑ In fast evolving environments such as humanitarian responses, the plan is reviewed more frequently and progress reports completed at shorter intervals.

Taking the standard to the next level (good practice):

- ☑ Oxfam leadership teams make safeguarding a recurring item for discussion in the agenda of their meetings.
- ☑ Processes are established for Oxfam teams to report on progress of safeguarding to leadership and governance bodies.
- ☑ An annual learning exercise takes place at the end of each year involving all the relevant stakeholders, eg. Oxfam teams, leadership and partners, to reflect on how the annual safeguarding plan has been implemented and what needs to be adjusted.

Evidence Checklist:

- ☐ An annual plan on safeguarding is in place and resourced.
- ☐ Safeguarding progress reports completed at least once every 6 months or more frequently as required.

2. Trained Safeguarding Focal Points are present in all Oxfam offices

All Oxfam teams identify and empower Safeguarding Focal point(s) to support the implementation of One Oxfam Safeguarding Policies. This will be proportionate to the scale of operations and related level of risk.

Why is this standard important?

The role of the Safeguarding Focal Point (SGFP) is an important safeguarding measure as it provides a local and trusted person that staff and communities can easily contact to seek advice and discuss any safeguarding concerns. The SGFP is a champion who plays an essential part in promoting good practice and helping teams work towards the One Oxfam Safeguarding Core Standards. SGFPs may also support senior management in coordinating the implementation of Oxfam's safeguarding policies and procedures.

Putting the standard into practice (minimum requirements):

- ☑ All Oxfam teams nominate at least two SGFP's ensuring gender balance. At least one SGFP is part of the program team to embed safeguarding within programs and partnerships.
- ☑ SGFPs are identified based on the scale of operations and related level of risks (eg. field offices and project locations, number of staff and projects, number of partner organisations).
- ☑ If required, Oxfam teams seek the support of Affiliate Safeguarding Leads and Regional Safeguarding Advisors to identify appropriate SGFPs.
- ☑ At least one member of the humanitarian team receives additional safeguarding training to act as a safeguarding focal point in the event of a humanitarian response.
- ☑ The candidates identified have a clear understanding of sexual harassment, exploitation and abuse risk factors (such as gender and power inequalities) and a commitment to prevention within Oxfam. They also have a clear understanding of child safeguarding issues. They are able to maintain strict confidentiality with commitment to follow procedures and have a mature attitude combined with good judgement & self-awareness.
- ☑ The SGFP signs a Terms of Reference (ToR) which describes their roles and responsibilities and undergoes a comprehensive safeguarding training programme to build their capability. The SGFP will receive ongoing support and mentoring from the Regional Safeguarding Advisors and Affiliate Safeguarding Leads.

Evidence Checklist:

- ☑ A minimum of two SGFP's are in place with clear Terms of Reference (ToR) and training
- ☑ Documented rationale for the selection of SGFPs (eg. mapping of scale of operations, level of risk etc).
- ☑ Training and capacity building plan for the SGFPs and training records.

Taking the standard to the next level (good practice):

- ☑ A suitably qualified paid Safeguarding role (eg. Safeguarding Coordinator) is recruited to coordinate safeguarding work across the team and to support SGFPs.
- ☑ Oxfam leadership enables the SGFP's to dedicate adequate proportionate time as agreed in the ToR to the SGFP function eg. 20% of their time.
- ☑ The SGFPs develop a coordination strategy that outlines the ways of working together as a team supported by leadership.

3. The Oxfam Code of Conduct is signed by all employees and related personnel

All Oxfam employees and related personnel read, understand, sign and abide by the relevant Oxfam Code of Conduct.

Why is this standard important?

A Code of Conduct outlines standards of acceptable and unacceptable behaviour for Oxfam employees and related personnel. The Code of Conduct protects the organisation and its personnel by providing guidance on how to avoid situations that may be perceived as exploitative, abusive or harmful to anyone, particularly children and young people. It also provides Oxfam, as an employer, with a sound basis to conduct disciplinary action in the case of breaches.

Putting the standard into practice (minimum requirements):

- ☑ Ensure all Oxfam employees and related personnel read, understand and sign the Oxfam Code of Conduct and that the signed Code of Conduct is held in HR files or as appropriate.
- ☑ Where required, translate the Code of Conduct into local languages and display the Code of Conduct in all offices, sub-offices and project locations.
- ☑ Have a copy of the Code of Conduct accessible to all employees and related personnel (eg. through intranet/website).
- ☑ Provide an induction and training to all personnel on the Code of Conduct and how it relates to the One Oxfam Safeguarding policies.
- ☑ For rapid humanitarian deployments, ensure all employees and related personnel receive a briefing on the Oxfam Code of Conduct, including professional behaviour standards in relation to children and young people within 48 hours of deployment.

Taking the standard to the next level (good practice):

- ☑ Develop context and culturally appropriate and accessible communication material explaining acceptable and unacceptable behaviour described in the Code of Conduct.
- ☑ Team meetings or other spaces are used for open discussions on the Code of Conduct and how it applies to Oxfam personnel and activities, including where there is contact with children and young people.
- ☑ Develop child and youth friendly Code of Conduct for programs/activities involving children and young people.

Evidence Checklist:

- ☑ Code of Conduct signed by all employees and related personnel and stored securely
- ☑ The attendance records of the online/offline/face to face induction on Code of Conduct.

4. Safe recruitment and screening processes are in place

Preventive measures are built into recruitment and screening processes to strengthen safeguarding.

Why is this standard important?

Safe recruitment and screening processes are essential to enable Oxfam to engage employees and related personnel who understand and adhere to Oxfam’s commitment, values and behaviour standards on safeguarding. Preventive measures in recruitment and screening strengthen external barriers through preventing access to vulnerable people by those with a known history of sexual exploitation and abuse. These measures will assist in deterring people who may target organisations that have weak or inconsistently applied procedures. It is important to apply safe recruitment and screening standards outlined below for all employees and related personnel as they are representatives of Oxfam in the community.

Putting the standard into practice (minimum requirements):

- ☑ Ensure all position advertisements contain key messages on safeguarding and Oxfam’s zero tolerance to Sexual Exploitation, Sexual Abuse and Sexual Harassment and Child Abuse.
- ☑ Assess all positions for the level of risk in relation to contact with communities and ensure that positions working directly with communities, especially children and young people, are subject to the highest level of screening such as behavioural-based interview questions.
- ☑ Ensure that all interviews include at least one question related to safeguarding, where appropriate with scenario or case studies.
- ☑ Require a minimum of two (documented) reference checks for all preferred candidates including targeted questions on the suitability of the applicant to work with communities. See Oxfam Global Referencing Framework Guideline for more details.
- ☑ Undertake criminal/police record check for all preferred candidates. Where there are challenges to obtain a criminal record check, provide a statutory declaration or local legal equivalent outlining efforts made to obtain a criminal record check and disclosing any charges and spent convictions related to Sexual Exploitation, Sexual Abuse and Sexual Harassment and Child Abuse.
- ☑ Employment contracts and/or Terms of Reference for all employees and related personnel contain provisions for disciplinary action, including dismissal, in relation to safeguarding concerns.

Evidence Checklist:

- ☑ Job descriptions state key safeguarding messages.
- ☑ Interview and written test templates include safeguarding questions, scenarios or case studies.
- ☑ For each selected applicant, the HR file contains the criminal record or alternative check, and documented reference checks.

Taking the standard to the next level (good practice):

- ☑ Potential applicants have access to the One Oxfam Safeguarding policies and Code of Conduct when applying for a position (attach the documents to the position description or make them available on the website).
- ☑ Interview panels have gender balance and includes staff that have received safeguarding training.

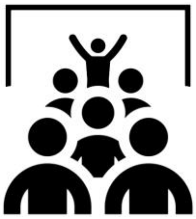

5. Regular and appropriate Safeguarding training is provided to all employees and related personnel

Safeguarding training is tailored to the local context and to specific roles and functions and is provided at regular intervals.

Why is this standard important?

Providing initial and refresher trainings on safeguarding is essential to build a collective and common understanding of One Oxfam Safeguarding policies. This is critical for employees and related personnel to internalise the specific roles and responsibilities they have as well as the values and behaviour expected from them as representatives of Oxfam.

Putting the standard into practice (minimum requirements):

- ☑ All personnel receive safeguarding training within their probation period or within the first 3 months, with refresher training every 2 years.
- ☑ The trainings are drawn from Oxfam training modules and adapted to the context. They cover Oxfam's safeguarding policies, Code of Conduct and the roles of the Safeguarding Focal Points (SGFP) and Safeguarding Teams.
- ☑ Specific safeguarding training sessions are organised to assist personnel to understand how different aspects of safeguarding are relevant to their role, programs and activities.
- ☑ The trainings contain exercises that explain how employees and related personnel can implement the policies and internalise them in their work given their specific function/role in the organisation.
- ☑ The trainings are delivered by personnel who have a comprehensive understanding of safeguarding. Support can be sought from Regional and Affiliate Safeguarding teams or external experts (e.g. partnering with a local child focused organisation to deliver Child Safeguarding training for Oxfam staff and partners).
- ☑ The leadership team actively promotes a safeguarding training plan, with dedicated resources to build the capacity of all employees and related personnel. Managers play a key role in allowing time for employees and related personnel to attend and deliver trainings.

Evidence Checklist:

- ☑ Annual training schedule that includes safeguarding trainings for new starters and refreshers throughout the year.
- ☑ Agendas,attendance records and materials used in training.
- ☑ Training (evaluation) reports/documentation.

Taking the standard to the next level (good practice):

- ☑ Measure and document the training impact on the level of understanding, internalisation and compliance to standards, through appropriate evaluation methods.
- ☑ Enable collaboration between the SGFPs and Gender, Protection and HR teams (and other relevant teams) to raise awareness through debates, thematic talks, lunch time events and safeguarding sessions at key staff meetings or during retreats to raise staff awareness. These informal mechanisms will foster common understanding of the gender and power dynamics and root causes of gender based violence and intersection with safeguarding within the communities where Oxfam works.

6. Safeguarding reporting and responding procedures are in place in all offices

Contextualised, clear, survivor-centred procedures are in place for reporting and responding to Safeguarding concerns.

Why is this standard important?

An effective safeguarding system requires a clear and well socialised incident reporting and handling procedure. Setting up effective mechanisms for reporting and responding to a safeguarding concern foster personnel and community trust and enable reporting. Failing in this area deeply undermines the efforts in strengthening the safeguarding system, exposing the survivor and the organisation to additional risks.

Putting the standard into practice (minimum requirements)

- ☑ Oxfam teams develop a safeguarding reporting procedure that is appropriate for their context. This is widely available and understood by employees and related personnel.
- ☑ Oxfam teams ensure their safeguarding reporting procedure meets One Oxfam policy requirements and is aligned to the One Oxfam Standard Operating Procedures also considering local laws. (*See also,* Annex.2 of the One Oxfam Child Safeguarding Policy, Reporting Misconduct SOP and One Oxfam Safeguarding Case management SOP).
- ☑ Oxfam teams map the legal framework and the services available for survivors (medical, psychosocial, legal and other forms of support) within their respective country and project locations; keeping this information up to date.
- ☑ All employees and related personnel are familiar with the safeguarding reporting procedure so they know how to report any sexual exploitation, sexual abuse and child abuse concerns.
- ☑ Oxfam safeguarding reporting and response procedures are explained to partners and integrated into partner agreements.
- ☑ Oxfam teams immediately report any suspected or alleged instances of sexual exploitation, sexual abuse and child abuse and One Oxfam safegarding policy non-compliance to the relevant Executing Affiliate.
- ☑ All safeguarding concerns, allegations and incidents are responded to in a timely manner, as per the One Oxfam Safeguarding Case management SOP and with a survivor centred approach[3].

Taking the standard to the next level (good practice):

- ☑ Integrate information on safeguarding reporting procedures throughout project and organisational communication materials.
- ☑ Document learning and recommendations from safeguarding case management procedures; communicate to relevant stakeholders to improve practices, procedures and reporting mechanisms.

Evidence Checklist:

- ☑ Documented reporting procedures for sexual harassment, exploitation, sexual abuse and child abuse concerns in line with One Oxfam SOP's.
- ☑ Updated mapping of services and local legal framework.
- ☑ Communication materials on how to raise a safeguarding concern (eg. Reporting flow charts, training materials/records).

[3] A survivor centred approach recognises and prioritizes the rights, needs and wishes of the survivor.

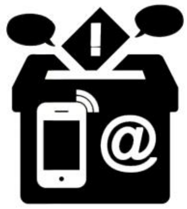

7. Community based feedback and complaint mechanisms are established

Safe, confidential and accessible feedback and complaint mechanisms are established for raising safeguarding concerns and are always functional; personnel and communities are aware and know how to use them.

Why is this standard important?

Feedback and complaint mechanisms ensure that communities have a voice, breaking down barriers to reporting and reducing the power imblance. Raising community awareness on Oxfam safeguarding commitments empowers communities to know their rights when interacting with Oxfam, and understand the behaviour expectations of Oxfam employees and related personnel.

Putting the standard into practice (minimum requirements):

- ☑ Consult with local staff, partners and community members to seek their perspectives on safeguarding risks which they may face and options for effective feedback and complaint mechanisms.
- ☑ Actively engage with PSEA networks where they exist or consult with other NGOS, UN agencies, government entities and seek their local experience, especially if there are existing feedback and complaints mechanisms in the project location.
- ☑ Working with partners and MEAL teams, set up different safe, confidential and accessible mechanisms for community members to give feedback or raise safeguarding concerns.
- ☑ Develop and test key messages on safeguarding - in local language with the project/field staff and community members involved in the project. Materials show clear messaging about acceptable and unacceptable behaviour from Oxfam employees and related personnel, and ways in which safeguarding concerns can be reported.
- ☑ Develop a child and youth friendly feedback and complaints mechanism, where relevant.
- ☑ Clear protocols established for identifying and confidentially triaging safeguarding complaints to EA safeguarding teams to respond appropriately.
- ☑ Proactive awareness raising activities in the community promoting key messages on safeguarding through relevant communication material and channels (eg. posters, leaflets, radio programmes, street theatre and focus group discussions). Communications are in local language and are culturally sensitive.
- ☑ Reporting lines are clear in case of disclosure of incident by community members during awareness raising activities.

Taking the standard to the next level (good practice):

- ☑ In community consultations, seek out people who may not provide their perspectives on safeguarding at community meetings or similar forums eg. people with disability, the elderly, children, LGBTIQ people.
- ☑ Staff in charge of set-up and monitoring of feedback and complaints mechanisms receive additional briefing on safeguarding and how to identify, respond to and report safeguarding concerns.
- ☑ Conduct regular learning exercises to identify how to improve the feedback and complaint mechanisms and document good practices.

More information available at:

[Setting up community feedback mechanisms in Oxfam programs : Guidance document]

Evidence Checklist:

- ☑ Safe and functional feedback and complaint mechanisms set up in project locations and documented.
- ☑ Communication materials on feedback and complaint mechanisms.
- ☑ Documentation of stakeholder consultation about the development of mechanisms.

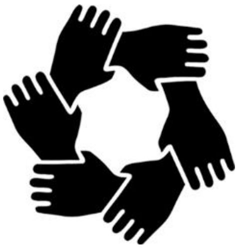

8. Partners adhere to One Oxfam Safeguarding Policies and Safeguarding Core Standards

Oxfam's Safeguarding policies and core standards are clearly communicated to partners; Partners are supported to develop and/or implement their own Safeguarding policies and procedures.

Why is this standard important?

The duty of care to keep communities safe is a shared responsibility between Oxfam and its partners; however Oxfam remains accountable for the risks that are associated with the activities implemented by its partners. Oxfam needs to partner with organisations that demonstrate commitment to safeguarding. Where there are gaps in partners' safeguarding practices, Oxfam has the responsibility to work with partners to strengthen their safeguarding system.

Putting the standard into practice (minimum requirements):

- ☑ Oxfam teams use the One Oxfam Partner Capacity Assessment or equivalent to assess and document whether partner's PSEA, youth and child safeguarding policies and practices meet Oxfam's Safeguarding Core Standards.
- ☑ If gaps are identified, develop a plan with the partner to address the gaps. The plan includes awareness and training activities for partner staff, support on case management, support on developing safeguarding policies and ongoing risk assessment of the activities. Monitor the plan regularly.
- ☑ Oxfam partner contracts or equivalent contain clauses that clearly articulate Oxfam's safeguarding requirements.
- ☑ All partners sign the Oxfam Partner Code of Conduct. Partner organisations can use their own Code of Conduct where it is assessed as aligned with Oxfam Partner Code of Conduct.
- ☑ Oxfam teams will provide regular trainings on One Oxfam Safeguarding policies and Safeguarding Core Standards to all partner organisations.
- ☑ Where required, support is sought from Regional and Affiliate Safeguarding teams. Oxfam teams can also collaborate with external experts to conduct safeguarding trainings with partners.
- ☑ Draw from Oxfam training modules to ensure consistency of content and make necessary adaptations to fit the local context.
- ☑ If monitoring indicates that a partner organisation is unable or unwilling to meet Oxfam's Safeguarding Core Standards after providing consistent support, Oxfam initiates measures to terminate the partnership.

Evidence Checklist:

- ☑ Documented partner capacity assessments that include an assessment of partners' safeguarding polcies and practices.
- ☑ Safeguarding clauses in partner agreements, MoUs and contracts.
- ☑ Signed Partner Code of Conduct equivalent.
- ☑ Copies of partner safeguarding policies and procedures.

Taking the standard to the next level (good practice):

- ☑ Work with partners from a strengths based approach to map out what practices exist in their organisation in terms of PSEA, child and youth safeguarding. Discuss any gaps or risks and include recommended measures in their policy and procedures. Document and share good practice examples from partners' safeguarding work.
- ☑ Organise training and learning sessions to work through a safeguarding risk assessment with the partner on the program/project. Use the risks and strategies identified to plan what the partner's policy and procedures should cover.
- ☑ Develop a shared understanding with your partners of safe behaviour standards for the local context and incident reporting processes.

9. Safeguarding risk assessment and management practices are established:

Safeguarding risk assessment and management practices are in place for all Oxfam offices, projects and activities. Safeguarding risk assessments are undertaken for projects at the design phase and updated at regular intervals.

Why is this standard important?

Safeguarding risk assessment is an effective preventive measure to keep everyone associated with Oxfam's work safe. Purposefully unpacking activities using a safeguarding lens will enable timely identification of potential safeguarding risks and their effective mitigation. Oxfam has a duty to ensure that a safeguarding risk assessment of its projects and activities is completed and safeguarding risks are monitored throughout the program cycle. Failing to mitigate these risks can expose people we work with, Oxfam employees and related personnel and the organisation to serious risks.

Putting the standard into practice (minimum requirements):

- ☑ A safeguarding risk assessment is conducted at the design and planning phase by unpacking all the relevant project activities. A comprehensive plan is developed to mitigate the identified risks. The project manager holds responsibility for completing the safeguarding risk assessment for the project.
- ☑ For ongoing projects past the design phase, a safeguarding risk assessment is completed as a priority.
- ☑ For each project, the safeguarding risks are identified and categorised as low, medium, high or extreme risks based on likelihood and impact. Mitigation steps to reduce or remove these risks are documented and personnel responsible to implement each mitigation step is identified.
- ☑ In addition to specific project activities, the safeguarding risk assessments consider factors such as the level of contact with communities, the range of people involved in delivering the project and challenges in monitoring the partners' safeguarding practices.
- ☑ The mitigation actions are monitored regularly to assess effectiveness and to identify any new, emerging or unexpected safeguarding risks. If there are significant changes to the project activities, an updated safeguarding risk assessment is conducted, alongside the implementation of new mitigation measures.
- ☑ If any high and extreme risks continue for a significant length of time, they are transferred to the organisational risk register and monitored by senior management and governance bodies.

Taking the standard to the next level (good practice):

- ☑ Undertake safeguarding risk assessments using participatory methods (eg. workshops) that involve the Oxfam project team, partners and community representatives.
- ☑ All high and extreme risks identified in the safeguarding risk assessment are moved into the relevant 'risk register' or equivalent. The project manager develops a 'risk management plan' for each high and extreme risk.
- ☑ Coordinate with the Monitoring, Evaluation and Learning teams for ongoing monitoring of high safeguarding risks as part of project monitoring.

Evidence Checklist:

- ☑ Completed safeguarding risk assessment and mitigation plan at project level.
- ☑ Documentation that safeguarding risk assessments are reviewed and updated regularly across the project cycle.
- ☑ Documentation of completed mitigation activities.

10. Procedures for handling images and personal information are established

Procedures are in place while collecting, storing and using images and personal information relating to communities as part of Oxfam's work.

Why is this standard important?

Oxfam collects and uses images and has access to personal information during the delivery of its work for multiple purposes. Individuals and communities we work with are at risk of sexual exploitation and abuse through various online platforms including; taking photographs, conducting interviews and community consultations, using images and quotes in documents and facilitating visits by external stakeholders to project sites (e.g. journalists, supporters and donors). Private and sensitive information about people we work with, including children, can easily be disseminated without informed consent, impacting people's right to privacy, dignity and safety. Oxfam employees and related personnel use social media for professional and personal matters creating potential for blurred boundaries and further risk of sexual exploitation and abuse (eg. Facebook, WhatsApp, skype, Instagram etc.).

Putting the standard into practice (minimum requirements):

- ☑ Oxfam teams follow Oxfam Ethical Content Guidelines and Oxfam Digital Safeguarding Policy relating to photographing, filming and using images and video of project participants and communities, gathering stories and conducting interviews.
- ☑ All program data (eg. MEL data) is collected and used in accordance with Oxfam Responsible Program Data Policy.
- ☑ Informed consent is obtained as per the steps outlined in Oxfam Ethical Content Guidelines. This is also applicable in cases where community members, including children and young people, are involved in Oxfam research or evaluations.
- ☑ Safeguarding risks related to content gathering and MEL activities are included in project level safeguarding risk assessments and appropriate mitigation measures implemented.
- ☑ Identifying information about people, especially children and young people, is never used in Oxfam documents, website and other communication material (eg. in a research report, in photo credits etc.).
- ☑ Use of ICT to communicate with community members is monitored by several staff, including management, and is set up using professional accounts only (eg. Facebook page is set up using work email account).

Taking the standard to the next level (good practice):

- ☑ Use child friendly informed consent forms when working directly with children and young people.
- ☑ Procedures for external visits (eg. media, donors) are in place, especially in emergency contexts to reduce safeguarding risks to communities through interviews or articles in TV, internet and social media.

Evidence Checklist:

- ☐ Relevant guidelines and policies (eg Ethical Content guidelines) are accessible to employees and related personnel.
- ☐ Signed informed consent forms stored securely.
- ☐ Safeguarding risk assessments include risks relating to content gathering, MEL and research activities.
