# Supplementary material for: Public knowledge, practices, and awareness of antibiotics and antibiotic resistance in Myanmar: The first national mobile phone panel survey
Source: PLoS One. 2022 Aug 18;17(8):e0273380. doi: 10.1371/journal.pone.0273380 (PMC9387831; doi:10.1371/journal.pone.0273380)
Supplement: Attachment — Submitted filename: gd-privacy-and-reporting-protocol-CATI-130521-en.pdf [file pone.0273380.s006.pdf]

Privacy and reporting protocol for safeguarding, protection and safety – CATI: EXAMPLE

Protocol to be prepared with researchers involved in gathering the data, safeguarding focal point and protection officer

Preamble

This example protocol is **not adequate in cases where the interview is about prevalence of violence**. Specific protocols have to be put in place when gathering data on prevalence of violence through face-to-face interview. UNWomen advises not to ask these questions through phone interviews.

This example protocol needs to be **adapted to the context** in which the interviews are taking place. For this, we recommend consulting the researchers involved in gathering the data, the safeguarding focal point and the protection officer as well as Oxfam or partner staff who are involved in gathering the contact information. The example below should be amended as required to **maximize safety** in the context at hand. In particular, safeguarding and protection staff will advise on the appropriate reporting channels and ways to access direct support available for survivors (survivors' centres or local clinics for example). In the example below, the researchers/interviewers are in charge of reporting, while the safeguarding and protection teams handle the referral process to institutions providing direct support.

Definitions:

- **Safeguarding**: Safeguarding in Oxfam is a set of procedures, measures and practices to ensure that Oxfam upholds its commitment to prevent, respond to and protect individuals from harm committed by staff and related personnel. In Oxfam, we focus on Sexual Exploitation, Sexual Abuse, Sexual Harassment (SEAH) and child abuse. We do this by: (i) listening to those who are affected; (ii) responding sensitively and safely when harm or allegations of harm occur; and (iii) learning from every case.
- **Protection**: Sexual exploitation, abuse, harassment or violence towards children and adults (where the perpetrator is not a member of Oxfam staff or partner staff).

Enabling privacy to maximize safety

Privacy can be understood in two ways: the right to privacy in the handling of one's personal data and the assurance of private set-up during the interview itself. Oxfam's Responsible Data Management Training pack, Going Digital 4 and 6,[1] cover the former in detail for the whole data cycle. This protocol focuses on **enabling a private set-up during the interview**, which includes **building on existing trust relationships, ensuring the interviewee is in a private environment during the call** (e.g. that no one is listening in the background) and **consenting for transmission of personal data**.[2]

---

[1] https://policy-practice.oxfam.org/resources/responsible-data-management-training-pack-620235/ https://policy-practice.oxfam.org/resources/going-digital-privacy-and-data-security-under-gdpr-for-quantitative-impact-eval-620884/ https://policy-practice.oxfam.org/resources/going-digital-computer-assisted-telephone-interviewing-cati-lessons-learned-fr-621188/

To conduct phone interviews, the research team needs to have access to names and phone numbers that may have been gathered by other persons within the same organization or a different one.

- **Contact from Oxfam staff or partner organization that gathered the contact information** in the first place and has an **established relationship** with potential interviewees.
    - Check that the person reached is indeed the person on the list (as people may have changed phone numbers since the personal data was collected).
    - Make clear when the contact information was gathered and for what purpose.
    - Ask for explicit verbal consent from the interviewee to take part in an interview which will be carried out by [add names of researchers].
    - Ask for explicit verbal consent from the interviewee to share their contact information with [add names of researchers].
    - Check if the phone number is shared with other household members. If so, ascertain whether the interviewee is happy for the researchers to let other household members know that they called, in case another household member picks up the phone instead when the researcher calls
        - If the interviewee is not comfortable with that situation, inform the interviewee that it will not be safe to do the interview and thank them for their time. As a consequence, their personal information will not be shared with researchers.

- **Contact from researchers with those who agreed to participate** (in previous step):
    - Pre-call from researcher to explain about the interview, ask them to set up a time when they can be alone.
        - If they have strong preference to be with someone, then the sensitive questions should be skipped.
        - If someone else picks up the phone (or if it is someone else's phone), ask to talk to the person you are trying to reach and for their personal phone number if they have one.
    - Set up a safe word (to be agreed upon as a team in advance) for the interviewee to use while the interview is ongoing to indicate that someone has come into the room. Introduce it at the beginning of the interview, and make it clear that once the interviewee says the safe word, the researcher will change the topic, stay on the phone for a few minutes, then reschedule the interview.
    - What if it is **not possible to speak with the intended interviewee**? For example the interviewee's partner does not want to let them answer the phone or wants to do the interview themselves
        - Carry out a very short version of the interview (excluding all sensitive questions and more) – integrated into survey design using skip logic.

Reporting protocols

The interview form is **not** the appropriate channel to report any misconduct. Two different reporting protocols are in place for use depending on who is involved in the misconduct. Researchers have a duty to report the following:

---

[2] Without the obtaining of such consent, the personal data cannot be transmitted to a third party. This will depend on: 1. the consent form used to gather the personal data in the first place; 2. whether the researchers are part of the same organizations who gathered the data. If they are not, specific data protection measures need to be put in place that go beyond this protocol. See the resources mentioned in footnote 1.

- Experiences of sexual exploitation, abuse, harassment, child abuse by a **member of Oxfam staff or partner staff** and/or related personnel (volunteer, community committee member, supplier, etc.)
  - If researchers are made aware of such a situation, they should write to [Add contact details based on conversation with safeguarding focal point]
  - [Add here what will happen to the information (for example, *the information will then be passed onto the safeguarding team,* so that the interviewers know what will happen and can communicate it to the interviewee]
  - Researchers **do not** need to ask questions about what had been told to them; it is not incumbent on the interviewer to then 'interview' the victim/survivor. Researchers are not investigating and cannot show any judgement against the person. The interviewee might, however, want to tell the interviewer more information, and so the interviewer should be prepared for this as much as possible. The researcher should respond sensitively and thank the interviewee for sharing.
  - Researchers have a duty to report to the Oxfam Safeguarding Team and should inform the interviewee as follows: "I am really concerned about what you have told me. I am going to ask that somebody from Oxfam speaks to you. This will be someone whose role is dedicated to safeguarding, that is to respond to and protect individuals from harm committed by staff and related personnel. They will not do anything before having talked to you."
  - If the person does not want their contact details to be shared, the researcher should still report but keep the interviewee's identity anonymous.

- Experiences of sexual exploitation, abuse, harassment or violence towards children and adults (the perpetrator is not a member of Oxfam staff or partner staff).
  - If researchers are made aware of such a case, they should remind interviewees that they can always contact the Oxfam hotline [Add contact number] and that if they do so, Oxfam's protection team will refer them to the right support institutions.
  - They should check with the interviewee whether they would prefer for the researcher to directly inform Oxfam Protection team: "I am really concerned about what you have told me. Would you like me to inform Oxfam Protection Team, so that they refer you to the institutions that can provide support for you. I would need to communicate your name and phone numbers to the Protection Team, as well as describe what you have just told me. Would you like me to do so?"
  - If the interviewee consents, the researcher will contact Oxfam Protection Team:
      - Name: [To be added, as agreed with Protection Team]
      - Phone number: [To be added]
      - Email: [To be added]
    - Information to share with Oxfam Protection Team if consent was given:
      - Name
      - Phone number
      - A brief description of what was shared with researcher – Researcher should **not** probe, only take the information as given.
  - Also, researchers should not store any of the referrals data. Papers used for collecting the data should be destroyed using a cross-cut shredder, as well as soft copy deleted.

In addition, researchers should always remind interviewees of the different channels to contact Oxfam and to report confidentially. The message below will pop-up as part of the script.

**Post-interview messaging for researchers – To be shared with all interviewees at the end of the interview and integrated at the end of the SurveyCTO script**
I would like to remind you that you can contact Oxfam for any issue you would like to discuss: [Add the number].<br><br>In addition, if you would like to report confidentially any matter, you can contact:<br><br>• The Oxfam Safeguarding Team: safeguarding@oxfam.org.uk or: +44 (0)1865 473813<br><br>• Oxfam's Whistleblowing Service: whistleblowing@oxfam.org.uk or: Arabic 00441865 47 2121<br><br>Researcher: please remember that you have a duty to report any safeguarding matter you have been made aware of, and you can do it confidentially with Oxfam Safeguarding Team via [Add contact details as agreed with safeguarding focal point]

Finally, support to researchers should be made available because of the emotional work provided by researchers, on the receiving end of difficult and traumatic stories.

- Oxfam free and confidential counselling line is available for Oxfam employees
- Other support mechanisms should be put in place for non Oxfam employees
