# Supplementary material for: Public knowledge, practices, and awareness of antibiotics and antibiotic resistance in Myanmar: The first national mobile phone panel survey
Source: PLoS One. 2022 Aug 18;17(8):e0273380. doi: 10.1371/journal.pone.0273380 (PMC9387831; doi:10.1371/journal.pone.0273380)
Supplement: Attachment — Submitted filename: review-NMMH.docx [file pone.0273380.s003.docx]

This study is important for baseline data for the antimicrobial use and antimicrobial resistance in Myanmar. As this study is the first nationwide data, there are some considerations especially methodology I want to point out.

Title: Public knowledge, practices, and awareness of antimicrobials and antimicrobial resistance in Myanmar: The first nationwide mobile phone panel survey

But the authors stated their aim of the study as "this study aimed to assess public knowledge and practices concerning antibiotics, as well as awareness of antibiotic resistance, to provide baseline evidence and identify gaps related to the deployment of strategic awareness-raising activities in Myanmar". Moreover, the questionnaires are asked about antibiotics only.

For non -expert readers, I think some clarifications are needed in the terms of antimicrobial or antibiotics (or) changing the title by using antibiotics only

**Introduction**

The authors should include other previous evidences from Myanmar even though they are not nationwide. From that, international audience can estimate the prevalence and burden of the interesting issue in Myanmar and the authors can explain the results in discussion.

**Method**

Before going to details, the authors should follow STROBE (for cross sectional study) reporting guideline especially for the method section because this is the most important part to decide the validity and reliability of the study.

Sample size:

line 85 to 94: The authors stated the steps to get the required sample size 1940. But there is difference what the reference book stated. According to the reference, Mobile phone panel survey in developing countries by world bank, in page 24, sample size calculation <https://openknowledge.worldbank.org/bitstream/handle/10986/24595/9781464809040.pdf?sequence=2&isAllowed=y>, the required sample size will be 3876 for 6 layers of stratifications (urban/rural, male/female, Younger/older). That is only for 2 layers of age stratification. If you have more layers, it will be increased according to the layers.

Please refer to the above link and justify the required sample (or) only mention another appropriate sample size calculation techniques.

Questionnaires:

Line 101 to 104: The authors stated the pretest was done. If any item analysis was done, please stated the results which will increase the validity of the questionnaires. Moreover, the study was claimed as nationwide study and to get baseline information, the authors should express detail procedure of development of the questionnaires and testing the internal and external consistency. I

Usually, the questionnaires contain general information of the study population as part A. I believe the authors already had that part. If so, please mention it and the data must show in the result section also. Because the readers want to know the general information of the study population.

According to the question A1, the population will divide 2, Yes and no. Further questions are based on "Yes" sub population. It is not clear whether the authors apply questions B, C and D to all participants or only to those who answer to Yes in question A1. Please mention it clearly in the method section and data analysis section for any sub group analysis.

Data Analysis: Thank to the authors for considering the sampling weights to reduce the bias. But need to mention any sub group analysis and how to handle the nonresponse rate because non response rate is important for MPPS.

Since the authors expressed the difference between groups, I suggest to calculate multivariable analysis for confounder-adjusted estimates to minimize the bias and show the strength of association.

**Result**

Again, I recommend to stick the reporting guideline. Please give the characteristics of study participants like Age, gender, residence, education, occupation, socioeconomic status etc

Please indicate number of participants with missing data or non-response rate. Since this is a survey type study, the readers want to know the non-response rate.

I know the table 3,4 and 5 are representing the sub groups. I am not sure the table 6. Please specify the total number of each group in the heading of the tables and in the test also. It is confused the readers. It is better if the authors show the flow diagram of the number of the response of the participants. eg;

**Discussion**

The whole discussion is well written and can explain the result well. Recommendation is also appropriate. But it can change after the authors adjust the data analysis. As I mention before, more Myanmar contexts/studies should be discussed here.

**Strength and limitation of the study**

Line 325-338, the authors want to express why they use MPPS. So, the whole para graph should express in the introduction or beginning of the method.

The authors should address other important factors such as social and economic factors which influence the results. Any potential bias should be expressed here.
