# Supplementary material for: Public knowledge, practices, and awareness of antibiotics and antibiotic resistance in Myanmar: The first national mobile phone panel survey
Source: PLoS One. 2022 Aug 18;17(8):e0273380. doi: 10.1371/journal.pone.0273380 (PMC9387831; doi:10.1371/journal.pone.0273380)
Supplement: Attachment — Submitted filename: Reviewer comment_PONE-D-22-06745_24Apr2022.docx [file pone.0273380.s007.docx]

**Reviewer comment for PLOS ONE**

Date of review: 24 Apr 2022

Manuscript Number: PONE-D-22-06745

Article Type: Research Article

Full Title: Public knowledge, practices, and awareness of antimicrobials and antimicrobial resistance in Myanmar: The first nationwide mobile phone panel survey

Corresponding Author: **Shinsuke Miyano**, Ph.D., MSc, M.D. National Center for Global Health and Medicine: Kokuritsu Kenkyu Kaihatsu Hojin, Kokuritsu Kokusai Iryo Kenkyu Center, Shinjuku, Tokyo JAPAN

**Reviewer comment**

Summary of the research and overall impression

This manuscript presented to access the knowledge, awareness and practice of using antibiotics among general populations in Myanmar using MPPS. Methodology session is required to revise/ add more detail information in terms of sampling, study period, data collection approach and data management. Data analysis is stated clearly. The results session is clear but the discussion and conclusions are required to improved.

This study will benefit to the community and national AMR program to strategically implement the national AMR action plan. I would agree to publish this article for wider data dissemination purpose to the academic audience as well as useful for the uptake by the national program.

It is recommended to improve the English language for academic writing and publication. For example, the use of tense (verb) in the results and discussion sessions should be past tense. The terms should be consistent: “participants” or “respondents” or “citizens” throughout the paper. Similarly, the term “overuse” or “misuse” should be consistent. If “overuse” and “misuse” are different definition, please elaborate in methods session.

Discussion on specific area of improvement

Abstract

1. The information presented in the abstract is clear, concise and complete. Thank you. Please add how the participants were randomly selected from which sampling frame. It said “from the panel” – please elaborate more what is the “panel” represent for.

Introduction

2. Add the key findings from the 3 countries in SEA region (India, Indonesia and Thailand) – eg. the proportion of public awareness and understanding about AMR in these counties.
3. List the countries where the WHO AMR nationwide survey was conducted in the SEA region.
4. Mention additional information about the national AMR program in Myanmar and the action/ activities implementing for AMR strategic action plan.

Methods

5. Explain the sampling procedure – how the participants were chosen from the panel data nationwide.
6. Table 1 – please move it to Annex.
7. Clarify the “period of the study” and “study areas” included in the study where the participants were enrolled in the survey.
8. Suggest to mention about “data management”, such as data cleaning and checking, how the data was stored for such a certain period of time, data security measure, etc.
9. Add the information about non-response rate, which is important for mobile phone interviews using IVR at the call center.
10. Mention how the data quality check was conducted (in a real-time?) for IVR and mobile phone interviews. Any follow up questions if the data is incomplete or occurs with errors.

Ethical considerations

11. Line 126 – please double check the name of the institutional board for ethical review. I assume it should be “Institutional **Review** Board”, not “Institutional **Research** Board”.

Results

12. Move the study period to the methods session.
13. The sample size (in the methods session) said 1940 participants, but how it was ended up with 2045 participants. Please explain in the methods session and in results session, total sample size is enough to present.
14. Line 133 – 68% lived in **RURAL** areas (according to the data from the table). Please correct.
15. Mention the proportion of participants in each state/ region in the Annex table.

Discussion and conclusions

16. Line 226 – “excellent representation” is not the academic writing; reflecting cannot measure how much “excellent” stands for. Please mention scientifically for the representativeness of the sample populations from the study.
17. In general, add the data from other literatures/ studies when explaining about the comparisons of data from this study findings, rather than mentioning “less likely” or “more likely”, so that the readers can get more detail understanding of how the differences is small or large in this study, compared to other studies.
18. Line 242-245 (…nationwide dissemination……multiple communication channels.) – how did you get this recommendation to be accomplished the knowledge dissemination in Myanmar. Please refer to and double check with the findings that can reference for this recommendation.
19. Line 266-267 (…in reality……without prescription.) – this statement is not included in the findings. Please add the reference if relevant or please remove the statement if that is not from the findings or no reference can be added. You may not want to add information without any evidence or reference in the discussion.
20. Line 309 – you cannot state “people from Myanmar” because this study was conducted in a sample population of Myanmar. You can state that “in this study, participants were less likely….”
21. Line 317-319 – suggest to mention that further collaboration with the agriculture, livestock and veterinary department for providing proper training and education program to the farmers and livestock, breeding farms regarding the AMR.
22. Limitation: mention the weakness of mobile phone survey, instead to face-to-face survey (eg. non-response rate).
23. Please mention anything about the safeguarding measures applied throughout the MPPS survey approach. If not, add this as the limitation.
24. However, the later part of the paragraph (line 327 onwards) stated about the advantage of MPPS. In this case, please present clearly “limitation” and “strength” of the survey. In addition, please also add the literature and reference about using MPPS methodology. Reference from Oxfam related to CATI (using phone interview) is provided for additional reference as necessary.

Conclusion

25. COVID-19 related AMR situation is just appeared in the conclusion. It is agreeable to include and discuss about this survey conducted before COVID-19 pandemic. Thus, include or move some information about COVID-19 related AMR situation in the discussion first, then present it again to conclude how this data could be impacted after the COVID-19 pandemic.
26. The facts in conclusion should be more concisely presented one by one, rather than general presentation.

Recommendation

I would like to propose the **major revision** for this manuscript.
