# Supplementary material for: Public knowledge, practices, and awareness of antibiotics and antibiotic resistance in Myanmar: The first national mobile phone panel survey
Source: PLoS One. 2022 Aug 18;17(8):e0273380. doi: 10.1371/journal.pone.0273380 (PMC9387831; doi:10.1371/journal.pone.0273380)
Supplement: Attachment — Submitted filename: AMR-Reviewer Comment.docx [file pone.0273380.s002.docx]

I am thankful to the editors and authors for giving me a chance to review this manuscript. It's overall well written by the results from a mobile phone panel survey among relatively large samples of the Myanmar population. For me, however, major improvements should be made on materials and methods and result sections to publish it in PlosOne.

**Abstract**

- Page 2, Line 33, the word “younger individuals” is misleading. Consider rephrasing or including the age range in years.

- Page 3, Line 39, a concluded sentence is unclear for me. What does it mean to “effective interventions”?

**Introduction**

- Introduction is relatively short and lacks detailed information concerning the AMR situation in Myanmar, the current healthcare system including challenges towards universal health coverage, and weaknesses in drug regulations, etc.

- It’d be better to include why authors chose the mobile phone panel for data collection and its efficiency referencing other literature or studies.

**Materials and Methods**

- Authors used MPPS as a data collection tool in this study. However, I believe authors should describe it in detail so that readers can understand easily about it.

- By a mobile phone panel, how did you ensure the involvement of similar gender, age, and residential area?

- Please include operational definitions of the variables (e.g., what does it mean “Urbanity”?)

- For data collection, given that mobile phone connections are a bit terrible in Myanmar, there might be some dropped out calls. How did you handle it?

- There might also be some people holding multiple numbers.

- We should have included routine QA and QC for collected data, if any.

- How did you validate the age of respondents by a call. Other family members could pick up phone calls rather than the owner of your intended number. Amid an increase in the number of dialers by some companies to promote their products or services, people nowadays avoid picking up such calls.

- How did you verify the translation of the questionnaire is correct and concise (e.g., back-translation method)?

- Authors mentioned making modifications to some questions after the pretest. What were those? Did you run any statistics to test its validity and reliability? Please consider attaching the Questionnaire in Burmese as a support file.

- Questionnaire was composed of four sections. How about general characteristics such as age, gender, and residential areas of respondents?

- Describes full name when it appeared first (e.g., SPSS)

- Line 117 – Authors mentioned about 95% CIs, but I did not find it in the later part.

- Please refer to the correct name of the IRB board from Myanmar @ <https://irbdmr.gov.mm/>.

**Results**

- Analysis is a kind of thin. I have observed that there were inequalities in the residential areas of the respondents. How did the authors make an account of such bias?

- In Table 1, there were some open-end questions (e.g., Q-C4). However, I did not find detailed results under the result section.

- Please include footnotes under each table as relevant (e.g., degree of freedom for Chi-square test, differentiations whether Fisher Exact or Chi-square is used for each variable)

- Age range (mean, min and max) for the respondents should be included. Some older people might have a problem in answering the phone or they are dependent on other family members who are usually taking care for their medications.

**Discussion**

- This part looks vast by including many things from the result section. I would suggest revising it in concise form, consider adding more about authors insights reflecting current situation in Myanmar.

- One other limitations of this study would be lacking information from provider side (e.g., Knowledge of pharmacist/ pharmacy). Normal person might be remote from A/ AMR awareness, at least, most respondents went to clinics, medical stores, or pharmacies for buying medicines rather than seeking self-treatment.

**-** Line 306-307, consider rephrasing the sentence to reflect current COVID-19 preventive measures in Myanmar.

**Other minor comments**

- Please recheck the format of the references, e.g., 1, 3, 7, 12, 14, 15.

- Ensure language errors, e.g., line 85 “High-frequency panel data nationwide.”
